# Quality over quantity: biopsy-anchored CT radiogenomics models outperform all-lesion training in a multi-tumour cohort despite a smaller sample size

**DOI:** 10.1007/s00330-026-12601-9

**Published:** 2026-05-16

**Authors:** Diana Ivonne Rodríguez Sánchez, Julian Middelkoop, Thera Vanneste, Olga Maxouri, Stephan Ursprung, Sajjad Rostami, Nino Bogveradze, Kalina Chupetlovska, Francesca Castagnoli, Federica Landolfi, Eun Kyoung Hong, Andrea Delli Pizzi, Nicolo Gennaro, Warissara Jutidamrongphan, Liliana Petrychenko, Petur Snaebjornsson, Zuhir Bodalal, Regina Beets-Tan

**Affiliations:** 1 Department of Radiology, The Netherlands Cancer Institute, Amsterdam, The Netherlands; 2 GROW Research Institute for Oncology and Reproduction, Maastricht University, Maastricht, The Netherlands; 3 Department of Radiology, University Hospital Tuebingen, Tuebingen, Germany; 4 Department of Radiology, American Hospital Tbilisi, Tbilisi, Georgia; 5 Department of Radiology, Royal Marsden Hospital, London, UK; 6 Division of Radiotherapy and Imaging, The Institute of Cancer Research, London, UK; 7 Radiology Unit, Sant’Andrea Hospital, Sapienza University of Rome, Rome, Italy; 8 Department of Radiology, Stanford University, Palo Alto, CA USA; 9 Department of Innovative Technologies in Medicine & Dentistry, G. d’Annunzio University of Chieti-Pescara, Chieti, Italy; 10 Institute for Advanced Biomedical Technologies, G. d’Annunzio University of Chieti-Pescara, Chieti, Italy; 11 Feinberg School of Medicine, Northwestern University, NMH/Arkes Family Pavilion Suite 800, 676 N Saint Clair, Chicago, IL 60611 USA; 12 Clinic of Radiology, Imaging Institute of Southern Switzerland (IIMSI), Ente Ospedaliero Cantonale (EOC), 6900 Lugano, Switzerland; 13 Department of Nuclear Medicine, Inselspital, Bern University Hospital, University of Bern, Bern, Switzerland; 14 Department of Pathology, The Netherlands Cancer Institute, Amsterdam, The Netherlands; 15 Faculty of Medicine, University of Iceland, Reykjavik, Iceland; 16 The Netherlands Cancer Institute, Amsterdam, The Netherlands; 17 Faculty of Health Sciences, University of Southern Denmark, Odense, Denmark; 18 Maastricht Radiation Oncology, Maastricht, The Netherlands

**Keywords:** Radiogenomics, Epidermal Growth Factor Receptor, Biopsy, Tumour heterogeneity, Machine Learning

## Abstract

**Objective:**

Radiogenomics aims to non-invasively predict tumour genotypes from imaging, but most studies assume molecular homogeneity by assigning a single biopsy-derived label to all lesions within a patient. This approach risks substantial label noise given well-documented interlesional heterogeneity. We investigated whether anchoring training to biopsy-confirmed lesions improves radiogenomic model performance and generalisability.

**Materials and methods:**

We retrospectively analysed 1646 patients (11473 segmented lesions) with contrast-enhanced CT and EGFR mutation status from next-generation sequencing at the Netherlands Cancer Institute, alongside an external NSCLC radiogenomics cohort (*n* = 158). All visible lesions were segmented, and the exact biopsy site was matched to its segmentation. Radiomic features were extracted, and machine learning models were trained with three lesion selection strategies: all lesions, non-biopsied lesions only, and biopsy-confirmed lesions only. To disentangle label quality from sample size, we created size-matched variants (one lesion per patient) for all-lesion and non-biopsied strategies.

**Results:**

All models achieved significant discrimination of EGFR status on internal validation (AUC = 0.62–0.68). However, performance of the all-lesion and non-biopsied models declined on external validation (AUC = 0.55–0.63), while the biopsy-anchored model maintained stable performance (AUC = 0.62), despite having only 1/10th of the training sample size. When training sets were size-matched, the biopsy-anchored approach significantly outperformed a model trained on all available lesions on external validation (*p* = 0.037).

**Conclusions:**

Radiogenomic models trained on biopsy-confirmed lesions outperform conventional all-lesion strategies in external validation, despite using an order of magnitude fewer samples. Prioritising lesion-level label fidelity can mitigate heterogeneity-driven noise, enhancing robustness and clinical translation of imaging-based genomic prediction.

**Key Points:**

***Question*** *Does assigning biopsy-derived molecular labels to all lesions introduce heterogeneity-driven label noise that reduces the generalisability of radiogenomic models?*

***Findings*** *Models trained exclusively on biopsy-confirmed lesions demonstrated superior external generalisability compared with all-lesion approaches, despite being trained on substantially fewer samples.*

***Clinical relevance*** *Biopsy-anchored radiogenomics improves the reliability of non-invasive mutation prediction by accounting for tumour heterogeneity, potentially supporting clinical decision-making when tissue sampling is limited or molecular results are discordant across lesions.*

**Graphical Abstract:**

**Figure:**
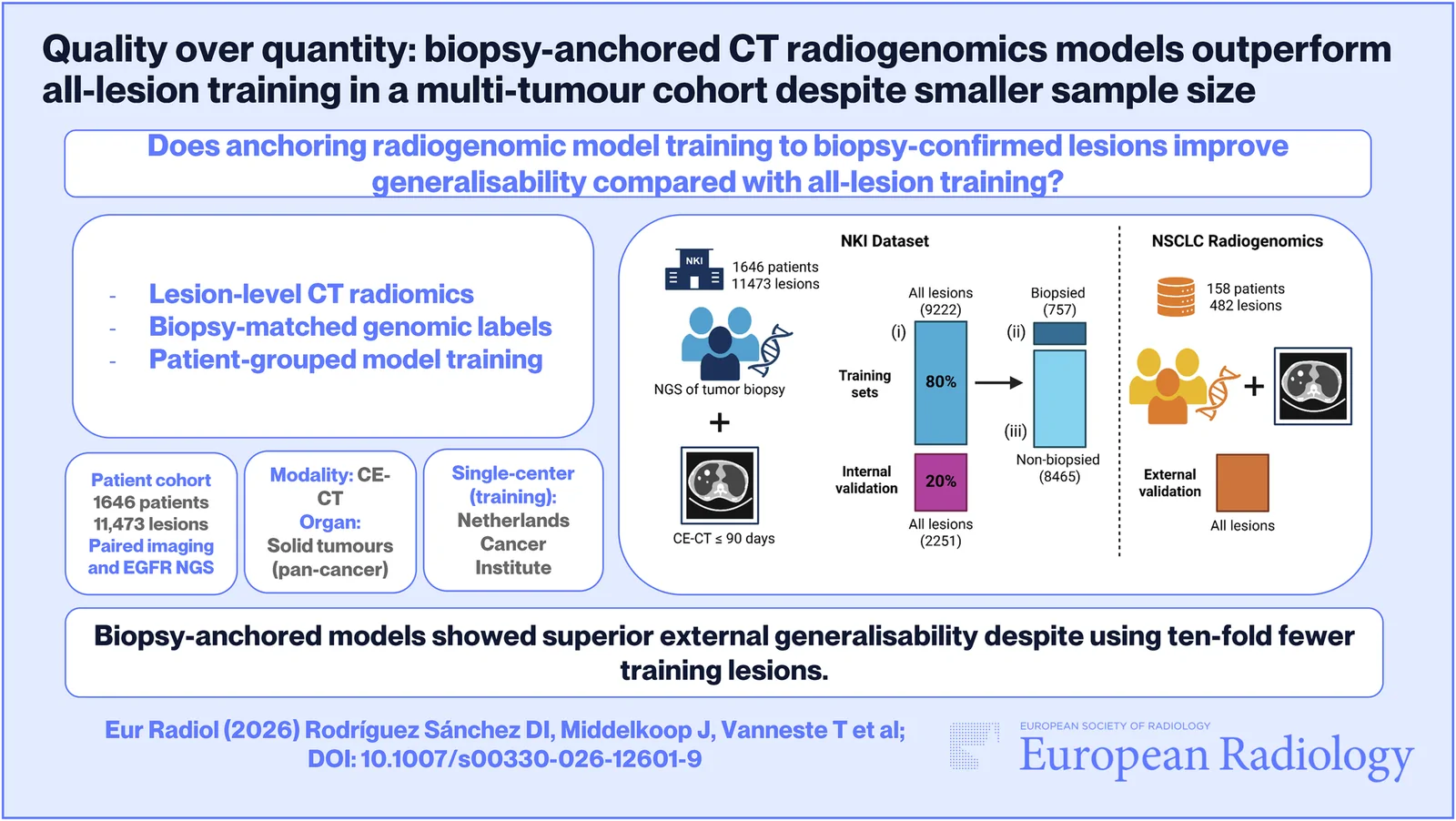

## Introduction

Precision oncology in solid tumours increasingly relies on the identification of oncogenic mutations that guide the use of targeted inhibitors or predict primary resistance. To date, these insights are almost exclusively derived from tissue samples obtained through biopsy or surgical resection. However, a single tissue sample offers only a limited view of a genomically complex malignancy [1]. Even within a single patient, spatially distinct lesions and subclones within the same lesion can harbour divergent mutations [2]. Such intra- and inter-tumoral heterogeneity means that the molecular profile derived from a single biopsy may not faithfully represent the biology of the disease elsewhere in the body. Sampling different lesions may therefore yield discordant mutation results, complicating genotype-driven therapy decisions.

The magnitude of this discordance, however, is not uniform across oncogenic drivers. Tumour suppressors such as tumour protein 53 (TP53) and common drivers such as Kirsten rat sarcoma viral oncogene homologue (KRAS) generally show high concordance between primary and metastatic sites, consistent with early clonal events and relative molecular stability. In contrast, other alterations exhibit substantially greater variability between lesions within the same patient. Activating mutations in the epidermal growth factor receptor (EGFR) represent a prominent example. A recent pan-cancer systematic review and meta-analysis of more than 15,000 paired samples reported a pooled EGFR discordance rate of approximately 16%, rising above 25% in previously treated patients and exceeding 30% in certain liquid-liquid comparisons [3]. These findings indicate that EGFR status cannot be assumed to be spatially uniform within a patient, particularly in advanced or treatment-exposed disease. This biological variability has driven interest in non-invasive strategies capable of capturing tumour biology across the entire disease burden [4].

Radiomics, which extracts quantitative descriptors of tumour morphology and texture from routine medical imaging [5, 6], has been applied to various clinical tasks including prognostication, treatment response prediction, and detection of occult disease [7–12]. Extending this concept, radiogenomics aims to connect imaging features with genomic and transcriptomic alterations [13, 14]. The core idea is that microscopic biological differences may be reflected in macroscopic imaging phenotypes, enabling genotype-phenotype relationships to be inferred non-invasively from routine scans [15]. Recent radiogenomic studies further suggest that specific oncogenic drivers, and their combinations, can give rise to distinct imaging phenotypes on CT, indicating that tumour genotype may shape macroscopic tumour morphology detectable on routine imaging [16]. Unlike tissue sampling, imaging covers all visible disease, offering the potential to map heterogeneity between lesions within the same patient [17].

Despite these advantages, most radiogenomic studies implicitly assume molecular homogeneity. Typically, a single “ground truth” label, derived from whichever lesion was biopsied, is applied to all lesions or imaging regions within a patient. However, in EGFR-driven tumours, lesion-to-lesion discordance occurs in approximately one out of six paired specimens overall and more frequently in treatment-exposed settings [3], meaning that this assumption can introduce significant label noise when biopsy-derived mutation status is assigned across all tumour lesions. In high-dimensional modelling, even modest rates of such label discordance can degrade signal, reduce reproducibility, and worsen the already known issues of overfitting and limited generalisability in radiomics research [18].

To our knowledge, no prior radiogenomic study has systematically evaluated whether restricting model training to biopsy-confirmed lesions, rather than including non-biopsied lesions or all lesions, improves external generalisability in the setting of lesion-level molecular heterogeneity. In this study, we address a central question: do CT-based radiogenomic signatures of EGFR represent a global patient-level phenotype or a lesion-local signal, and does enforcing lesion-level label fidelity improve out-of-sample performance?

To investigate this, we assembled a large multi-tumour dataset where every visible lesion on contrast-enhanced CT (CE-CT) was segmented and, importantly, the exact lesion used for tissue sequencing was tracked and matched to its segmentation. Using this information, we compared three data modelling approaches: training with all lesions, using only biopsied lesions, and using non-biopsied lesions. Generalisability was assessed through patient-aware cross-validation, an internal hold-out set, and an independent external cohort, with size-matched analyses to disentangle label quality from data volume. This design provides a rigorous test of whether fidelity-first training produces more reproducible and interpretable radiogenomics that can credibly map lesion-level heterogeneity. Establishing this evidence base lays the groundwork for radiogenomics to serve as a dependable complement to tissue testing in precision oncology.

## Materials and methods

### Study population and ethical approval

We retrospectively collected a consecutive series of patients at the Netherlands Cancer Institute (NKI) who had undergone tumour biopsy for next-generation sequencing (NGS) as part of routine clinical care. These patients were cross-referenced with our institutional Picture Archiving and Communication System (PACS) to identify those with a baseline CE-CT scan (acquired within 90 days of biopsy), minimising potential effects of tumour evolution or treatment. An external validation cohort was also included, derived from the publicly available Non-small-cell lung cancer (NSCLC) NSCLC-Radiogenomics dataset from The Cancer Imaging Archive [19, 20].

All candidate scans were independently reviewed by a team of radiologists to confirm adequate image quality, the presence of at least one visible tumour, and suitability for quantitative image analysis. Inclusion criteria required the presence of intravenous contrast enhancement and a reconstructed slice thickness of ≤ 5 mm. From the final curated cohort, clinical variables (age, sex, and tumour type) and EGFR mutation status (mutated vs. wild-type) were recorded.

Ethical approval was obtained from the institutional review board (IRBd19-147) of the NKI. All data collection and analysis were conducted in accordance with institutional policies and applicable regulations. Reporting in this study adhered to the CheckList for EvaluAtion of Radiomics research (CLEAR) criteria [21]. The code used for the analyses in this study is available via the following GitHub repository: https://github.com/nki-radiology/biopsy_anchored_radiogenomics.

### Image acquisition and segmentation

All included images were CE-CT scans acquired as part of routine clinical care. Patients fasted for 2–4 h prior to scanning; no bowel preparation was performed. Scans were performed on multidetector CT systems from Philips, Siemens Healthineers, GE Healthcare, or Canon Medical Systems, with detector configurations ranging from 16 to 320 channels (Supplementary Table 1). Iodine-based contrast agents were administered intravenously (90-130 mL of Omnipaque at 300 mg/mL), and portal venous phase images were captured approximately 70s after injection. Images were reconstructed with slice thicknesses ranging from 1 to 5 mm. The median in-plane pixel spacing was comparable for both cohorts, with 0.782 mm (IQR = 0.726–0.839 mm) for the NKI cohort and 0.777 mm (IQR = 0.703–0.820 mm) for the external validation cohort.

All NKI scans were reviewed by a board-certified radiologist (N.B.) to assess image quality, artefacts, and suitability for segmentation. Six board-certified radiologists (N.B., E.K.H., F.C., F.L., N.G., and A.D.P.; 4–8 years of experience) manually delineated all identifiable lesions using 3D Slicer (version 5.4.0), including primary tumours (when present), lymph nodes ≥ 10 mm in short-axis diameter, and all detectable metastases (with a maximum of 10 lesions per organ). Periodic team meetings were held to ensure consistency of delineation and to reach consensus via discussion on ambiguous cases. Following segmentation, two readers (K.C., S.U.; 5–7 years of experience) performed quality control and annotated each lesion by type and anatomical location.

The pathology report for each NKI case was reviewed to determine the recorded biopsy site and match it to the corresponding segmentation by one reader (S.U.), consulting prior imaging in PACS when necessary to confirm lesion identity. The external validation cohort was segmented with the same guidelines by a radiologist (W.J., 7 years of experience), and segmentation quality was verified by a second radiologist (L.P., 5 years of experience). All scans were anonymised using institutional pipelines and stored in Nearly Raw Raster Data (NRRD) format.

### Radiomic feature extraction and selection

Radiomic features were extracted using PyRadiomics v3.1.0 [22], an open-source library developed in accordance with the Image Biomarker Standardisation Initiative (IBSI) guidelines [23]. Prior to extraction, all CT images were resampled to an isotropic voxel size of 1 × 1 × 1 mm³ using B-spline interpolation to harmonise spatial resolution across scanners and acquisition settings. From each segmented lesion, 2016 features were computed, encompassing shape descriptors, first-order intensity statistics, and multiple classes of second-order texture features derived from grey level co-occurrence (GLCM), run length (GLRLM), size zone (GLSZM), dependence (GLDM), and neighbouring grey tone difference (NGTDM) matrices. Collectively, these features capture complementary aspects of tumour phenotype, including morphology, internal heterogeneity, and intensity distributions. Because image transformations (e.g., LoG, wavelets) significantly alter the dynamic range of voxel intensities, applying a fixed bin width across all image types might result in extreme variations in the number of discrete bins, leading to sparse matrices or loss of texture information. Therefore, we optimised the bin width independently for each filter type to consistently yield between 30 and 130 discrete grey level bins, an approach shown to maximise the reproducibility and robustness of texture features [24]. Detailed feature extraction parameters, including applied image filters, discretisation settings, and pre-processing steps, are described in Supplementary Methods 1. Feature definitions are available in Griethuysen et al [25].

Radiomic feature pre-processing (i.e., normalisation) and selection were performed exclusively on the training set and applied to test sets, to prevent any data leakage. Selection was achieved using an ensemble approach that combined six supervised techniques: recursive feature elimination, Pearson correlation, Chi-square testing, logistic regression, light gradient boosting machine, and random forest. Each method independently ranked features by their association with EGFR mutation status, and the top 100 features from each list were retained. Features appearing in at least four of the six lists were considered consensus-relevant and selected for model training. Using multiple complementary feature selectors reduces dependence on the biases of any single algorithm, improves stability of the selected signature [26, 27], and is well-suited for high-dimensional radio(geno)mic data [28–30]. To assess the independence of the selected radiomic features, we computed the absolute Pearson correlation coefficient between all feature pairs in the training cohort. A correlation matrix was constructed to visualise pairwise relationships, and the average absolute correlation was computed to evaluate overall feature redundancy.

### Machine learning modelling

For each patient, the biopsy site was identified from pathology reports and matched to the corresponding segmented lesion on baseline CT scans. When the biopsied lesion was no longer visible or segmentable on the earliest pre-treatment scan (for example, after resection or radiotherapy), patients remained in the study but were excluded from the biopsied-lesion cohort.

Patients were randomly divided into an 80% training set and a 20% internal validation set using a patient-aware strategy, ensuring that all lesions from the same individual were kept within a single partition. From the training cohort, three distinct lesion cohorts were created: (i) all segmented lesions, (ii) only the biopsied lesions, and (iii) only the non-biopsied lesions. To examine whether predictive performance was influenced by data volume or the content of per-lesion information, we designed two complementary analyses. In the first analysis, models were trained directly on their respective lesion cohorts, preserving the natural imbalance in lesion numbers. In this setting, the all-lesion and non-biopsied models were trained on considerably more data than the biopsied-lesion model (effectively ten-fold more samples), which typically included only a single lesion per patient. The second analysis controls for this imbalance by constructing size-matched versions of the all-lesion and non-biopsied cohorts. For each, ten independent sub-samples were generated by randomly selecting a single lesion per patient until the total number of lesions matched that of the biopsied-lesion group. This procedure ensured that all three lesion-selection strategies were trained on cohorts of equal size and similar per-patient representation.

**Fig. 1:**
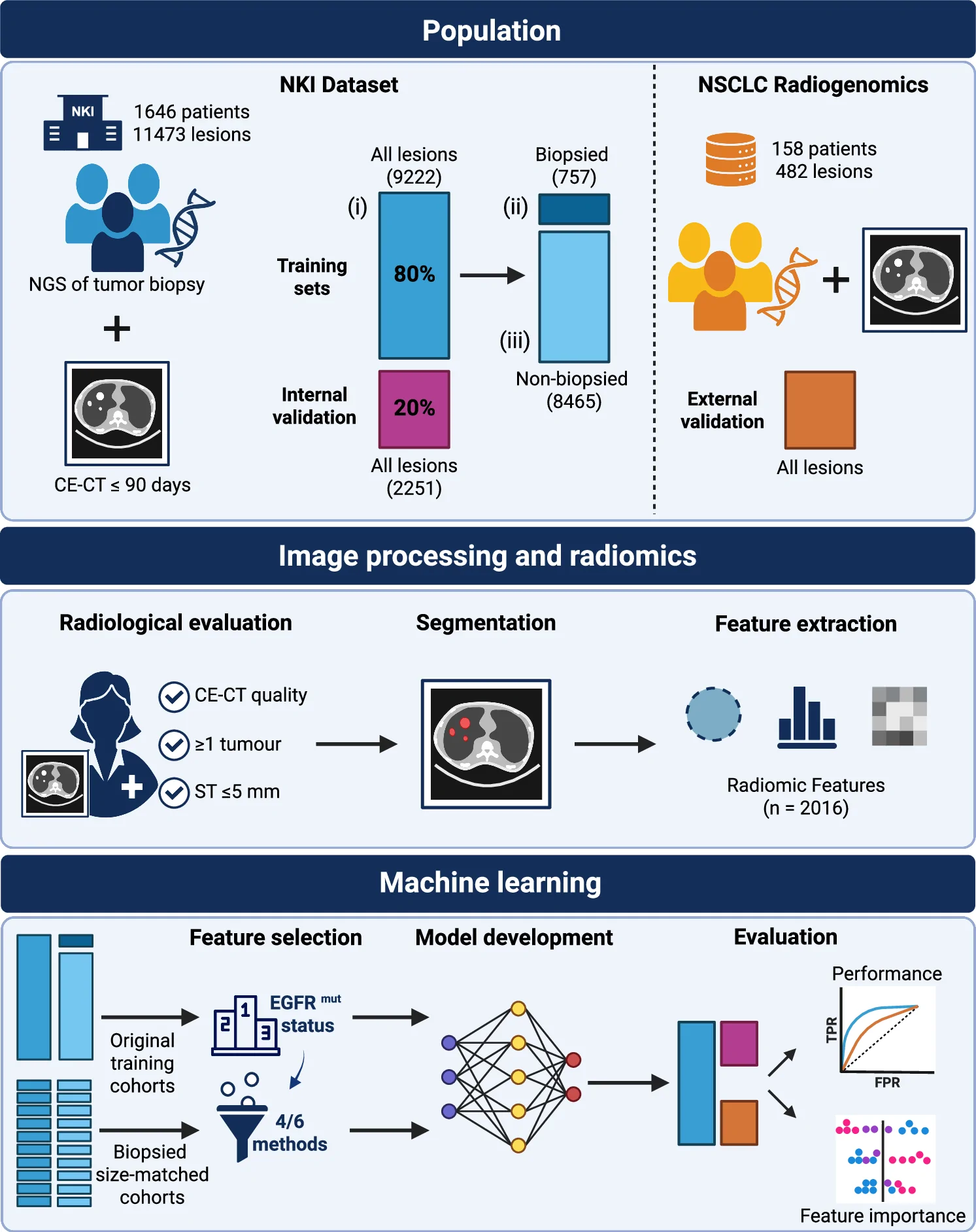
Study design overview. Schematic of the cohort construction, imaging and radiomics pipeline, and machine-learning workflow

Model development was performed using the Tree-based Pipeline Optimisation Tool (TPOT), an automated machine learning framework based on genetic programming [31–33]. Within the training set, TPOT implemented five-fold cross-validation with GroupKFold at the patient level, guaranteeing that lesions from the same patient were never split across folds. Each generation of the evolutionary algorithm assessed a population of 100 candidate pipelines with diverse classifiers and hyperparameter settings. The best-performing pipelines were iteratively recombined and mutated to form the next generation. This process was repeated for 100 generations, exploring > 10,000 candidate models. The final pipeline was retrained on the full training set, using class-balanced weights to mitigate class imbalance and ensure robust learning of patterns in the minority class. Finally, the trained model was evaluated on both the internal hold-out set and the independent external validation cohort. A schematic representation of the study design is shown in Fig. 1.

### Statistical analysis

Baseline clinical and demographic variables were compared between groups using non-parametric or categorical tests as appropriate. We tested the normality of the radiomic feature distribution using the Shapiro–Wilk test. Continuous variables were assessed with the Mann–Whitney *U*-test, while categorical variables were compared using the Chi-square test. The performance of each machine learning model was evaluated on both the internal validation set and the independent external validation cohort. Predictive accuracy was measured using the area under the receiver operating characteristic curve (AUC), accuracy, F1 score, positive predictive value (PPV), and negative predictive value (NPV). To derive measures of uncertainty, all performance metrics were calculated through 1000-repeat bootstrapping, with the median and 95% confidence intervals reported. Comparisons of AUCs between models were conducted using the method of Hanley and McNeil [34]. To explore model explainability, we calculated SHapley Additive exPlanations (SHAP) values for each trained model, which quantified the contribution of individual radiomic features to predictions. Multiple testing was corrected using the Benjamini-Hochberg approach. All analyses were performed in Python v3.12.9 using numpy (2.1.3), pandas (2.2.3), matplotlib (3.10.1), pydicom (2.4.4), pyradiomics (3.1.0), seaborn (0.13.2), scipy (1.15.2), sklearn (1.6.1), shap (0.48.0), statsmodels (0.14.5), tpot (0.12.2), trimesh (4.7.1), xgboost (3.0.0).

## Results

### Patient characteristics

From the NKI institutional database, 5094 tissue samples underwent NGS between 2015 and 2020. Of these, 3265 were excluded because no suitable CE-CT was available. Among the remaining patients, 1815 had CE-CT within 90 days of the biopsy. After further exclusions for technical or clinical reasons (e.g., non-delineable lesions, post-surgical imaging, artefacts), the final NKI cohort consisted of 1646 patients (1686 scans) with at least one segmentable tumour.

**Fig. 2:**
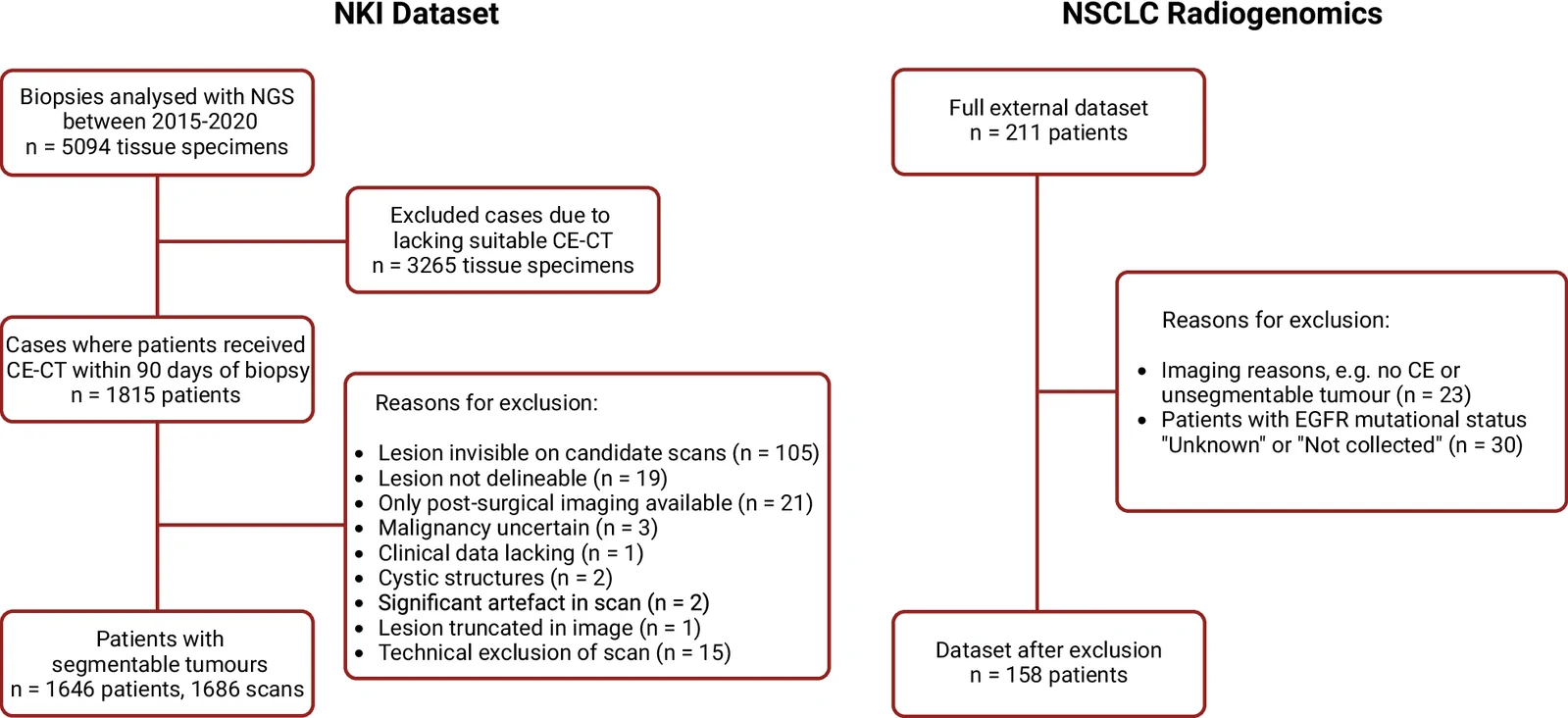
Cohort construction flowchart. Inclusion and exclusion diagrams for the internal NKI pan-cancer dataset and the external NSCLC Radiogenomics set, showing the screening process, application of imaging eligibility criteria, reasons for exclusion, and the final analysed populations

For the external validation set, the publicly available NSCLC Radiogenomics dataset contained 211 patients. After excluding 23 patients due to a lack of contrast enhancement and 30 more patients without EGFR mutational status, 158 patients were eligible. Detailed information on the inclusion and exclusion criteria workflow for both cohorts is provided in Fig. 2.

The NKI cohort was split at the patient level into 80% training and 20% internal validation. The test set consisted of 330 patients with 2251 segmented lesions, of which 181 were biopsied-confirmed. No significant differences were observed between the training and validation sets for age (*p* = 0.781), number of lesions per patient (*p* = 0.894), tumour type distribution (*p* = 0.270), or EGFR mutation rate (*p* = 0.090). Within the training set, three lesion-selection cohorts were created: the all-lesion cohort (9222 lesions from 1316 patients), the non-biopsied cohort (8465 lesions from 1101 patients), and the biopsied-lesion cohort (757 lesions from 751 patients). (Table 1) Cohort characteristics are described in Supplementary Table 2.

**Table 1:** Baseline characteristics of the analysed cohorts

	NKI data				External validation set
	Training set all lesions	Training set non-biopsied lesions	Training set biopsied lesions	Test set	
Patients (*n*)	1316	1101	751	330	158
Scans (*n*)	1347	1124	757	339	158
Total number of lesions (*n*)	9222	8465	757	2251	482
Age (median [IQR])	62.0 [53.0–69.0]	61.0 [53.0–69.0]	62.0 [54.0–70.0]	62.0 [54.0–69.0]	68.5 [64.0–75.8]
Lesions per patient (median [IQR])	4.0 [2.0–10.0]	5.0 [2.0–11.0]	1.0 [1.0–1.0]	4.0 [2.0–11.0]	2.0 [2.0–3.0]
EGFR mutation (*n*, %)					
Wild type	8083 (87.6%)	7412 (87.6%)	671 (88.6%)	2003 (89.0%)	317 (65.8%)
Mutated	1139 (12.4%)	1053 (12.4%)	86 (11.4%)	248 (11.0%)	165 (34.2%)
Tumour type (*n*, %)					
Lung cancer	3676 (39.8%)	3360 (39.7%)	316 (41.7%)	927 (41.2%)	482 (100%)
Colorectal cancer	2258 (24.5%)	2117 (25.0%)	141 (18.7%)	516 (22.9%)	-
Others	3288 (35.7%)	2988 (35.3%)	300 (39.6%)	808 (35.9%)	-

Summary of patient and lesion counts, number of scans, age, lesions per patient, EGFR mutation prevalence, and tumour-type distribution across the training cohorts, internal validation, and external validation set

### Feature selection

A total of 2016 radiomic features were extracted from each segmented lesion. Ensemble feature selection using six supervised methods identified nine consensus features in the all-lesion cohort, 12 in the non-biopsied cohort, and three in the biopsied-lesion cohort (Supplementary Table 3). Five features overlapped between the all-lesion and non-biopsied cohorts, while no features overlapped with the biopsied cohort. Across all three lesion cohorts, the selected features showed reasonable independence (Supplementary Figs. 1–3).

### Individual feature-level associations with EGFR mutational status

As the selected radiomic features were not normally distributed (Supplementary Table 4), their associations with EGFR mutational status were assessed in the internal validation using the Mann–Whitney *U*-test. For each feature, we additionally quantified effect size using Cohen’s *D*, calculated its individual discriminative ability by area under the ROC curve, and reported statistical significance of the difference between wild-type and mutated samples after correction for multiple testing.

Several selected features showed significant univariate differences between EGFR-mutated and wild-type lesions, with moderate effect sizes (Cohen’s *D* > 0.5) and AUCs approaching 0.65–0.67. Recurrent features such as square_glcm_JointAverage and square_firstorder_Median, were consistently associated with EGFR status. The three-feature biopsied-lesion signature also demonstrated significant associations, though with lower individual AUCs (adjusted *p*-values < 0.001, Table 2).

**Table 2:** Predictive capacity of selected features per training cohort

Feature name	Selected in which cohort	Cohen’s *D*	AUC	*U* statistic	*p*	Adjusted *p* (*q*)
Wavelet-LLH FirstOrder 90Percentile	All lesions	0.39	0.64	177,447	< 0.001	**< 0.001**
LoG_σ_5 FirstOrder Kurtosis	All lesions	−0.23	0.45	271,575	0.016	**0.019**
LoG_σ_5 FirstOrder Skewness	All lesions	0.22	0.54	226,570	0.024	**0.027**
Wavelet-HLL FirstOrder Skewness	All lesions	−0.02	0.48	259,672	0.242	0.248
Square FirstOrder Median	All lesions, Non-biopsied	0.51	0.64	178,429	< 0.001	**< 0.001**
Square GLCM JointAverage	All lesions, Non-biopsied	0.51	0.64	179,341	< 0.001	**< 0.001**
Wavelet-LLH GLSZM ZonePercentage	All lesions, Non-biopsied	0.46	0.63	183,149	< 0.001	**< 0.001**
Exp GLDM DNUN	All lesions, Non-biopsied	0.52	0.63	184,335	< 0.001	**< 0.001**
Original GLDM DNUN	All lesions, Non-biopsied	0.41	0.63	186,063	< 0.001	**< 0.001**
LoG_σ_1 GLRLM RunEntropy	Biopsied	0.50	0.65	174,424	< 0.001	**< 0.001**
Wavelet-LLH GLSZM SZNUN	Biopsied	0.39	0.63	181,726	< 0.001	**< 0.001**
LoG_σ_1 GLDM DependenceEntropy	Biopsied	0.20	0.59	205,352	< 0.001	**< 0.001**
LoG_σ_1 GLSZM ZonePercentage	Non-biopsied	0.52	0.66	171,278	< 0.001	**< 0.001**
Square GLCM LMC1	Non-biopsied	−0.52	0.36	319,605	< 0.001	**< 0.001**
Square GLSZM SZNUN	Non-biopsied	0.51	0.63	182,894	< 0.001	**< 0.001**
LBP GLSZM LGLZE	Non-biopsied	−0.36	0.40	298,091	< 0.001	**< 0.001**
Wavelet-HLH GLCM ClusterShade	Non-biopsied	−0.03	0.59	203,713	<0.001	**< 0.001**
Log GLSZM SAE	Non-biopsied	−0.35	0.43	284,026	< 0.001	**< 0.001**
LBP GLCM ClusterShade	Non-biopsied	−0.13	0.48	259,515	0.248	0.248

For each feature retained by the ensemble selector, the table reports the training cohort(s) in which it was selected, its effect size (Cohen’s *d*), univariate AUC, *U*-statistic, and multiple-testing-adjusted *p*-value

Bold values indicate statistically significant results

### Model performance across full cohorts

**Fig. 3:**
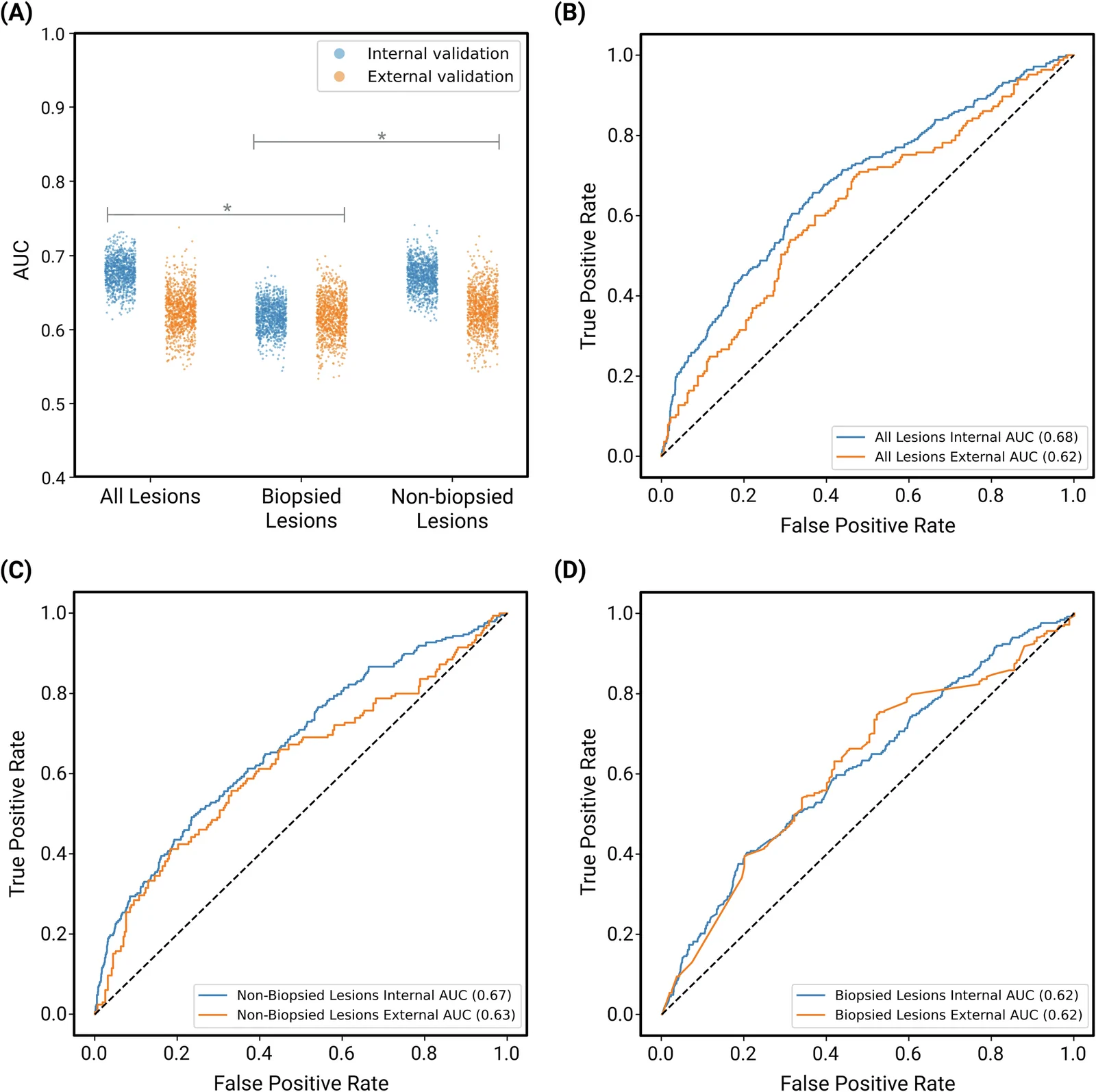
Performance of EGFR prediction models across different lesion-selection strategies. Internal and external discrimination of EGFR status for models trained on all lesions, biopsied lesions, and non-biopsied lesions. **A** Bootstrap distribution of AUC estimates for internal and external testing. **B**–**D** ROC curves for each model. Internally, the all-lesion and non-biopsied models performed slightly better (AUC 0.68 and 0.67, respectively) than the biopsied model (AUC 0.62), whereas externally, all three models converged around 0.62. Pairwise comparisons of AUC values were performed using the Hanley and McNeil method. *p < 0.05

Predictive models were trained for each lesion cohort and evaluated using five-fold cross-validation, followed by internal and external validation (Fig. 3A and Supplementary Table 5). During cross-validation, the mean AUCs were 0.69 for the all-lesion model, 0.67 for the biopsied-lesion model, and 0.71 for the non-biopsied model.

On internal validation, all three models achieved statistically significant discrimination of EGFR mutation status. The all-lesion model reached an AUC of 0.68 (95% CI: 0.64–0.72, *p* < 0.001), the biopsied-lesion model 0.62 (95% CI: 0.58–0.65, *p *< 0.001), and the non-biopsied model 0.67 (95% CI: 0.63–0.71, *p* < 0.001) (Table 3). In this setting, the all-lesion (*p* = 0.016) and non-biopsied (*p* = 0.037) models outperformed the biopsied-lesion. Furthermore, multivariate analysis revealed that the model’s predictions were independent of tumour type and lesion volume (Supplementary Table 6).

**Table 3:** Model performance on internal and external cohorts

Training cohort	Validation cohort	ROC-AUC	F1-score	Accuracy	PPV	NPV	*p*
All lesions	Internal	0.68 [0.64–0.72]	0.30 [0.25–0.34]	0.77 [0.76–0.79]	0.23 [0.19–0.26]	0.92 [0.91–0.93]	**< 0.001**
Biopsied	Internal	0.62 [0.58–0.65]	0.23 [0.20–0.26]	0.54 [0.52–0.56]	0.14 [0.12–0.16]	0.92 [0.90–0.93]	**< 0.001**
Non-biopsied	Internal	0.67 [0.63–0.71]	0.28 [0.24–0.33]	0.77 [0.76–0.79]	0.22 [0.18–0.26]	0.92 [0.90–0.93]	**< 0.001**
All lesions	External	0.62 [0.57–0.68]	0.52 [0.46–0.57]	0.52 [0.47–0.56]	0.39 [0.34–0.45]	0.75 [0.68–0.82]	**< 0.001**
Biopsied	External	0.62 [0.56–0.67]	0.78 [0.74–0.81]	0.65 [0.60–0.69]	0.66 [0.62–0.71]	0.40 [0.23–0.59]	**< 0.001**
Non-biopsied	External	0.63 [0.57–0.68]	0.50 [0.44–0.55]	0.52 0.48–0.57]	0.39 [0.34–0.45]	0.73 [0.67–0.79]	**< 0.001**

Discrimination and classification metrics with 95% bootstrap confidence intervals for the three full-cohort models, showing higher internal AUCs for all-lesion and non-biopsied training, convergence of all models on external validation, and consistently significant performance

Bold values indicate statistically significant results

However, the relative advantage of the larger cohorts diminished on external validation, where performance converged across models (Fig. 3B–D). The all-lesion model achieved an AUC of 0.62 (95% CI: 0.57–0.68, *p* < 0.001), the biopsied-lesion model 0.62 (95% CI: 0.56–0.67, *p* < 0.001), and the non-biopsied model 0.63 (95% CI: 0.57–0.68, *p* < 0.001). The performance of the all-lesion model declined significantly between the internal and external cohorts (*p* = 0.037), and the non-biopsied model also trended towards reduced performance (*p* = 0.117). In contrast, the biopsied-lesion model maintained stable performance across datasets and demonstrated non-inferiority compared with both the all-lesion (*p* = 0.500) and non-biopsied (*p* = 0.398) models on external validation, despite being trained on an order of magnitude fewer lesions.

### Performance of size-matched models

To account for differences in lesion numbers between cohorts, ten size-matched models were trained for both the all-lesion (Supplementary Table 7) and non-biopsied groups (Supplementary Table 8), each restricted to one lesion per patient and matched to the biopsied-lesion cohort size (*n* = 757). Clinical characteristics of the sub-sampled sets are provided in Supplementary Table 9.

After size matching, the performance of both the non-biopsied and all-lesion models decreased, whereas the biopsied-lesion model maintained its performance. For the non-biopsied models, the average cross-validated AUC during training was 0.71 (range: 0.66–0.74). On internal validation, the mean AUC was 0.64 (range: 0.58–0.67), and on the external validation, performance declined to 0.58 (range: 0.51–0.68, *p* = 0.039). Compared to models trained on all available non-biopsied lesions, predictive performance was reduced on both the internal (*p* = 0.141) and external (*p* = 0.100) datasets.

**Fig. 4:**
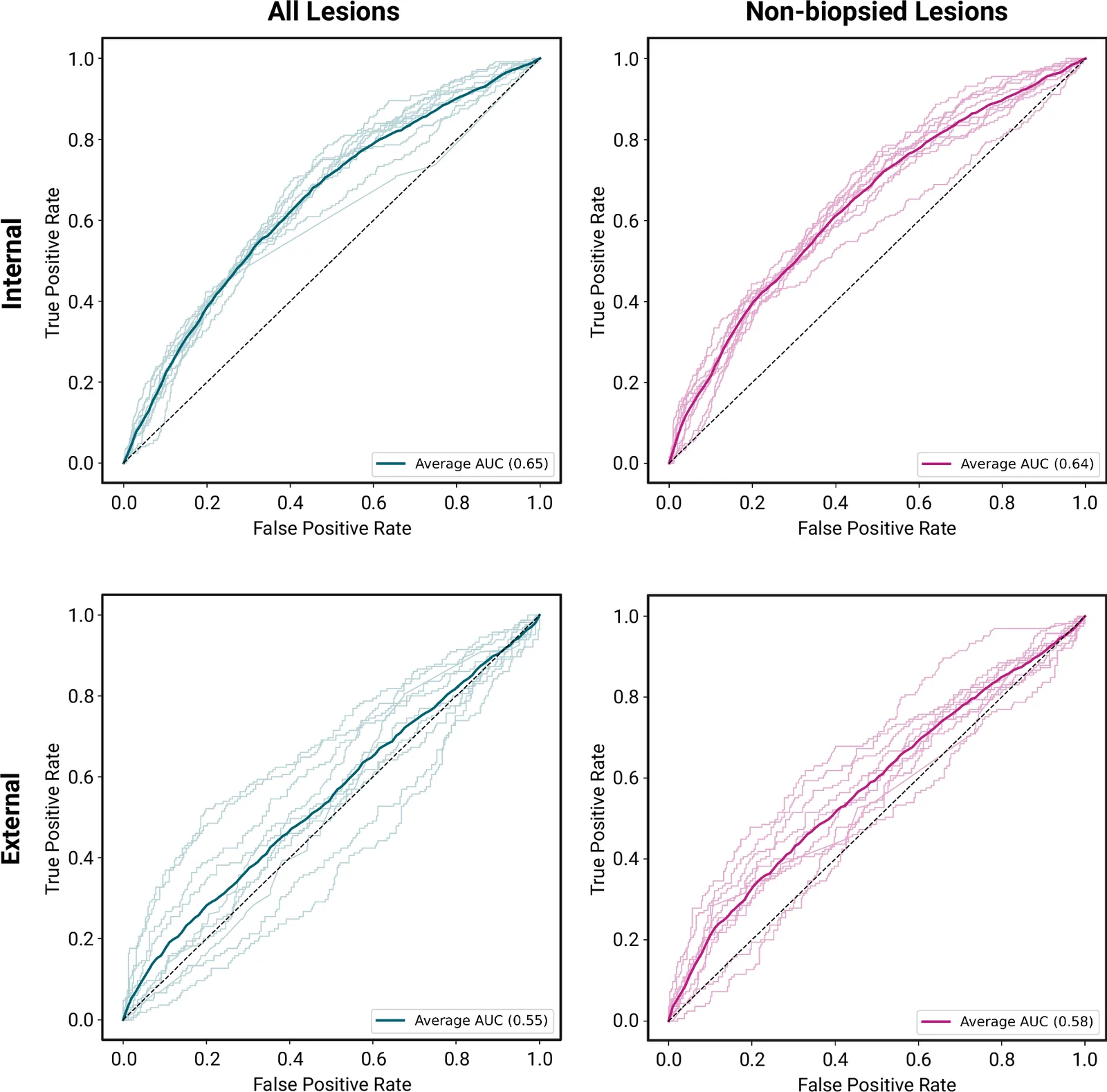
ROC-AUC curves of the size-matched models. Ten size-matched sub-samples per strategy, restricted to one lesion per patient and matched to the biopsied-lesion cohort size, demonstrate reduced performance when sample size advantages are removed

For the all-lesion models, the average cross-validated AUC during training was 0.70 (range: 0.64–0.76). On internal validation, the mean AUC was 0.65 (range: 0.59–0.68), which decreased to 0.55 (range: 0.39–0.68) on the external cohort (*p* = 0.002). Both internal (*p* = 0.140) and external (*p* = 0.037) performance were lower than when using all available lesions. Importantly, the average external performance of the size-matched all-lesion models was significantly worse than that of the biopsied-lesion model (*p* = 0.037). ROC-AUC curves for the size-matched models are shown in Fig. 4.

### Biopsied-lesion–only evaluation of full-cohort models

To test whether lesion-level label fidelity drives performance, we restricted the internal validation to only biopsy-confirmed lesions (*n* = 181, 18 EGFR positive) and evaluated the three classifiers trained on their full cohorts. The biopsy-anchored model was able to significantly predict EGFR mutational status, achieving an AUC of 0.67 (95% CI: 0.55–0.79, *p* = 0.018). The all-lesion and non-biopsied models showed weak non-significant predictive performance, with AUC = 0.53 (95% CI: 0.38–0.67, *p* = 0.678) and 0.56 (95% CI: 0.40–0.71, *p* = 0.392), respectively (Supplementary Table 10); (Tables 4 and 5)).

**Table 4:** Size-matched performance using non-biopsied lesions as training

Training cohort	Validation cohort	Sample	ROC-AUC	F1-score	Accuracy	PPV	NPV	*p*
Non-biopsied lesions	Internal	Sub-sample 1	0.64 [0.61–0.68]	0.27 [0.23–0.31]	0.77 [0.75–0.78]	0.21 [0.17–0.24]	0.92 [0.90–0.93]	**< 0.001**
		Sub-sample 2	0.63 [0.59–0.67]	0.26 [0.23–0.30]	0.73 [0.71–0.74]	0.19 [0.16–0.22]	0.92 [0.90–0.93]	**< 0.001**
		Sub-sample 3	0.65 [0.61–0.70]	0.00 [0.00–0.00]	0.89 [0.88–0.90]	0.00 [0.00–0.00]	0.89 [0.88–0.90]	**< 0.001**
		Sub-sample 4	0.66 [0.62–0.69]	0.26 [0.23–0.30]	0.61 [0.59–0.63]	0.17 [0.14–0.19]	0.93 [0.92–0.94]	**< 0.001**
		Sub-sample 5	0.66 [0.63–0.70]	0.26 [0.22–0.30]	0.69 [0.68–0.71]	0.18 [0.15–0.21]	0.92 [0.91–0.93]	**< 0.001**
		Sub-sample 6	0.64 [0.60–0.68]	0.26 [0.22–0.30]	0.75 [0.73–0.77]	0.19 [0.16–0.23]	0.91 [0.90–0.93]	**< 0.001**
		Sub-sample 7	0.58 [0.54–0.62]	0.24 [0.20–0.29]	0.72 [0.70–0.74]	0.17 [0.14–0.20]	0.91 [0.90–0.93]	**< 0.001**
		Sub-sample 8	0.64 [0.61–0.68]	0.25 [0.22–0.29]	0.69 [0.67–0.71]	0.17 [0.14–0.20]	0.92 [0.90–0.93]	**< 0.001**
		Sub-sample 9	0.67 [0.64–0.71]	0.27 [0.23–0.30]	0.64 [0.62–0.66]	0.17 [0.15–0.20]	0.93 [0.91–0.94]	**< 0.001**
		Sub-sample 10	0.65 [0.62–0.69]	0.25 [0.22–0.29]	0.66 [0.64–0.68]	0.17 [0.14–0.19]	0.92 [0.91–0.93]	**< 0.001**
		**Average**	**0.64**	**0.23**	**0.72**	**0.16**	**0.92**	
	External	Sub-sample 1	0.59 [0.53–0.65]	0.49 [0.44–0.53]	0.37 [0.32–0.41]	0.34 [0.29–0.38]	0.60 [0.47–0.73]	**0.002**
		Sub-sample 2	0.51 [0.45–0.56]	0.51 [0.46-0.56]	0.35 [0.31–0.40]	0.34 [0.30–0.39]	0.70 [0.40–0.92]	0.790
		Sub-sample 3	0.68 [0.63–0.73]	0.00 [0.00-0.00]	0.66 [0.61–0.70]	0.00 [0.00–0.00]	0.66 [0.61–0.70]	**< 0.001**
		Sub-sample 4	0.53 [0.47–0.59]	0.48 [0.44–0.53]	0.35 [0.31–0.39]	0.33 [0.29–0.38]	0.54 [0.39–0.70]	0.374
		Sub-sample 5	0.58 [0.52–0.63]	0.51 [0.46–0.55]	0.34 [0.30–0.39]	0.34 [0.30–0.39]	0.57 [0.14–1.00]	**0.005**
		Sub-sample 6	0.51 [0.46–0.57]	0.48 [0.43–0.54]	0.41 [0.37–0.45]	0.35 [0.30–0.40]	0.67 [0.57–0.76]	0.629
		Sub-sample 7	0.55 [0.50–0.60]	0.50 [0.46–0.55]	0.37 [0.33–0.42]	0.35 [0.30–0.39]	0.69 [0.54–0.83]	0.084
		Sub-sample 8	0.64 [0.58–0.70]	0.50 [0.46–0.55]	0.36 [0.32–0.40]	0.34 [0.30–0.39]	0.67 [0.47–0.86]	**< 0.001**
		Sub-sample 9	0.65 [0.59–0.71]	0.51 [0.46–0.56]	0.36 [0.32–0.40]	0.35 [0.30–0.39]	0.79 [0.53–1.00]	**< 0.001**
		Sub-sample 10	0.58 [0.53–0.64]	0.50 [0.44–0.55]	0.42 [0.37–0.46]	0.36 [0.30–0.41]	0.71 [0.62–0.80]	**0.003**
		**Average**	**0.58**	**0.45**	**0.40**	**0.31**	**0.66**	

Ten sub-sampled models trained on one lesion per patient, matched to the biopsied-lesion cohort size, with internal and external metrics and confidence intervals; performance decreased compared with the full non-biopsied model and fell significantly on external validation (mean AUC 0.58)

Bold values indicate statistically significant results

**Table 5:** Size-matched performance using all lesions as training

Training cohort	Validation cohort	Sample	ROC-AUC	F1-score	Accuracy	PPV	NPV	*p*
All lesions	Internal	Sub-sample 1	0.59 [0.55–0.63]	0.03 [0.01–0.06]	0.88 [0.87–0.89]	0.14 [0.03–0.31]	0.89 [0.88–0.90]	**< 0.001**
		Sub-sample 2	0.67 [0.63–0.70]	0.25 [0.21–0.28]	0.65 [0.63–0.67]	0.16 [0.14–0.19]	0.92 [0.90–0.93]	**< 0.001**
		Sub-sample 3	0.61 [0.58–0.65]	0.27 [0.22–0.30]	0.72 [0.70–0.74]	0.19 [0.15–0.22]	0.92 [0.91–0.93]	**< 0.001**
		Sub-sample 4	0.65 [0.61–0.68]	0.26 [0.23–0.30]	0.63 [0.61–0.65]	0.17 [0.14–0.19]	0.93 [0.91–0.94]	**< 0.001**
		Sub-sample 5	0.64 [0.60–0.68]	0.00 [0.00–0.00]	0.89 [0.88–0.90]	0.00 [0.00–0.00]	0.89 [0.88–0.90]	**< 0.001**
		Sub-sample 6	0.68 [0.64–0.71]	0.27 [0.23–0.30]	0.70 [0.68–0.72]	0.18 [0.15–0.21]	0.92 [0.91–0.93]	**< 0.001**
		Sub-sample 7	0.66 [0.62–0.69]	0.27 [0.23–0.30]	0.65 [0.63–0.67]	0.17 [0.15–0.20]	0.93 [0.91–0.94]	**< 0.001**
		Sub-sample 8	0.64 [0.61–0.68]	0.26 [0.22–0.30]	0.68 [0.66–0.70]	0.18 [0.15–0.21]	0.92 [0.91–0.93]	**< 0.001**
		Sub-sample 9	0.66 [0.63–0.70]	0.27 [0.23–0.31]	0.66 [0.64–0.68]	0.18 [0.15–0.20]	0.93 [0.91–0.94]	**< 0.001**
		Sub-sample 10	0.67 [0.63–0.70]	0.27 [0.23–0.30]	0.68 [0.66–0.70]	0.18 [0.15–0.21]	0.92 [0.91–0.94]	**< 0.001**
		**Average**	**0.65**	**0.22**	**0.71**	**0.16**	**0.92**	
	External	Sub-sample 1	0.52 [0.46–0.57]	0.35 [0.29–0.40]	0.61 [0.57–0.65]	0.36 [0.11–0.62]	0.66 [0.61–0.70]	0.513
		Sub-sample 2	0.68 [0.63–0.73]	0.52 [0.47–0.56]	0.63 [0.59–0.68]	0.35 [0.31–0.40]	0.80 [0.69–0.93]	**< 0.001**
		Sub-sample 3	0.43 [0.38–0.49]	0.49 [0.44–0.54]	0.36 [0.32–0.40]	0.34 [0.29–0.38]	0.60 [0.44–0.73]	**0.016**
		Sub-sample 4	0.51 [0.45–0.56]	0.51 [0.46–0.56]	0.35 [0.30–0.39]	0.34 [0.30–0.39]	0.80 [0.00–1.00]	0.768
		Sub-sample 5	0.58 [0.52–0.63]	0.50 [0.00–0.00]	0.66 [0.61–0.70]	0.00 [0.00–0.00]	0.66 [0.61–0.70]	**0.008**
		Sub-sample 6	0.55 [0.49–0.61]	0.48 [0.43–0.53]	0.38 [0.33–0.42]	0.34 [0.29–0.38]	0.62 [0.50–0.74]	**< 0.001**
		Sub-sample 7	0.52 [0.47–0.58]	0.57 [0.47–0.56]	0.35 [0.31–0.39]	0.34 [0.30–0.39]	1.00 [1.00–1.00]	**0.037**
		Sub-sample 8	0.67 [0.62–0.73]	0.53 [0.48–0.58]	0.45 [0.41–0.50]	0.38 [0.33–0.43]	0.81 [0.73–0.89]	**< 0.001**
		Sub-sample 9	0.39 [0.34–0.44]	0.27 [0.23–0.31]	0.39 [0.34–0.43]	0.34 [0.29–0.38]	0.85 [0.75–0.94]	**< 0.001**
		Sub-sample 10	0.61 [0.56–0.67]	0.50 [0.45–0.56]	0.54 [0.50–0.59]	0.37 [0.32–0.42]	0.73 [0.65–0.80]	**< 0.001**
		**Average**	**0.55**	**0.47**	**0.47**	**0.32**	**0.75**	

Ten sub-sampled models trained on one lesion per patient to match the biopsied-lesion cohort size, with internal and external results; performance was lower than the full all-lesion model and dropped significantly on external validation (mean AUC 0.55), performing worse than the biopsy-anchored model externally

Note: *p*-values for individual sub-samples are provided for descriptive transparency only. Because overall model evaluation relies on the pooled metrics rather than the statistical significance of individual sub-samples, multiple-testing correction was not applied

Bold values indicate statistically significant results

### Model interpretability with SHAP

**Fig. 5:**
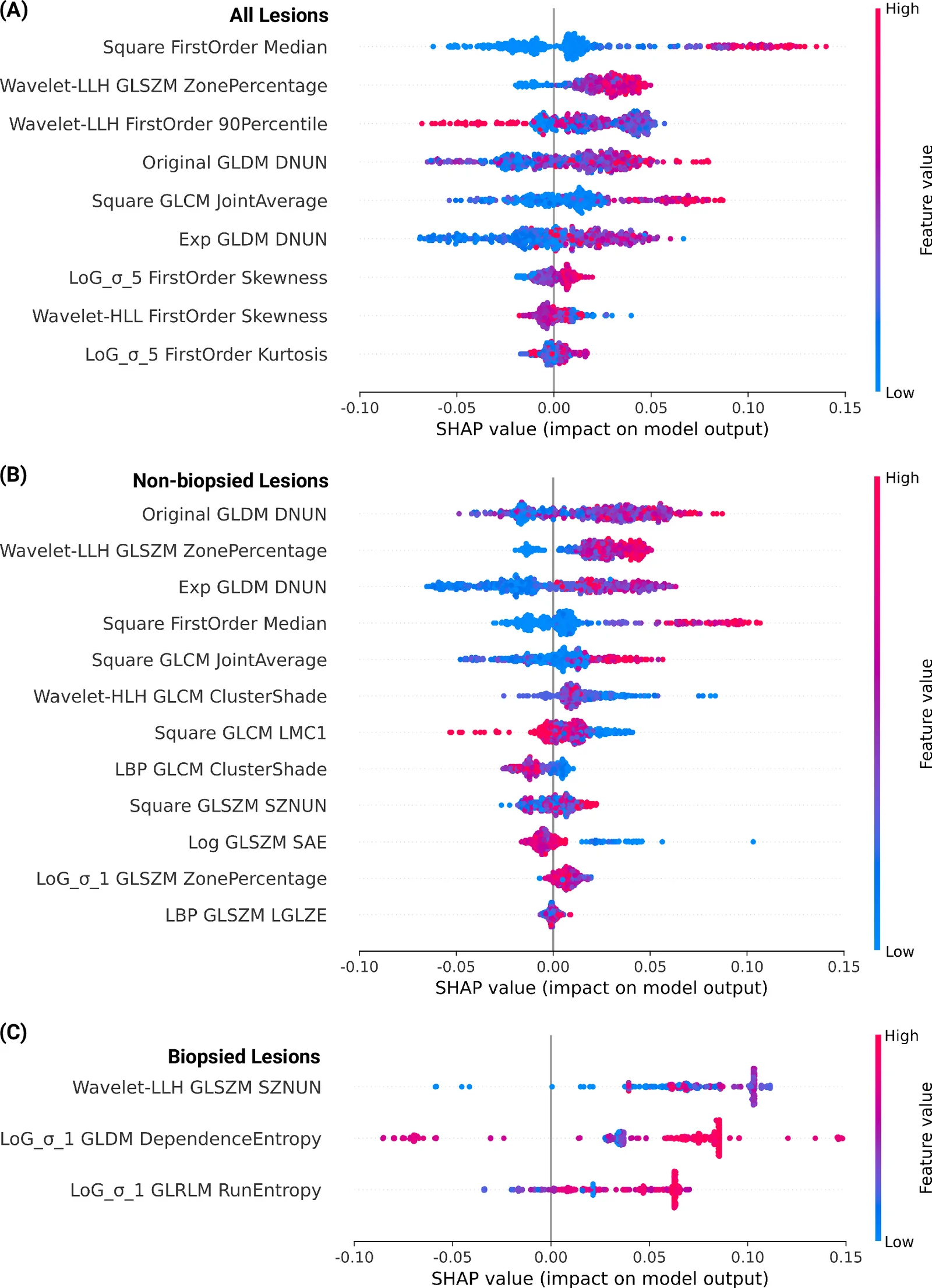
Model explainability analysis. SHAP beeswarm plots display the contribution of individual radiomic features to predictions across the three models. The all-lesion (**A**) and non-biopsied (**B**) models share impactful features such as Square FirstOrder Median and Wavelet-LLH GLSZM ZonePercentage that push predictions towards EGFR mutation at higher values. The biopsy-anchored (**C**) model relies on a compact, distinct three-feature texture signature

To better understand how individual radiomic features contributed to EGFR prediction, we computed SHAP values for each trained model. In the all-lesion model (Fig. 5A), predictive signal was distributed across a diverse set of texture and intensity features, with Square FirstOrder Median and Wavelet-LLH GLSZM ZonePercentage identified as the most contributory features; these were also among the top features in the non-biopsied model (Fig. 5B). The biopsied-lesion model (Fig. 5C) showed a distinct pattern, with contributions concentrated within its three selected features.

## Discussion

This multicentre study showed that a radiogenomic model trained on biopsy-confirmed lesions achieved similar or better performance than models trained on all detectable lesions per patient. All models showed modest discrimination on the independent test sets (internal AUCs ranging between 0.62 and 0.68), consistent with other EGFR prediction algorithms in the literature [35–37]. However, the biopsy-anchored model retained its predictive capacity in external validation, whereas the models trained on either all lesions or the non-biopsied tumours showed a substantial performance drop during external validation. When training sets were size-matched across strategies, the biopsy-anchored approach significantly outperformed the others on the external cohort (AUC = 0.62 versus 0.55 for the all-lesion model). These outcomes were achieved despite the anchored model being trained on only ~750 lesions, compared to more than 9000 for the conventional model. Together, these findings support that anchoring radiogenomic models to biopsy-proven lesions can enhance their generalisability without sacrificing performance.

Biologically, our results align with the understanding that metastatic tumours can be heterogeneous in genotype [38–40], hence treating all lesions as molecularly identical introduces potentially contradictory supervision during training. By restricting training to lesions with known mutations, we eliminate this source of label noise and present the model with a cleaner learning task. In high-dimensional machine learning, even relatively small proportions of mislabelled lesions can significantly impair classifier performance [41]. Our approach addresses this by design: each lesion’s CT features are paired with the correct mutation status (or excluded if unknown), thereby increasing the signal-to-noise ratio for associations between imaging phenotypes and genotype. In contrast, a traditional radiogenomic model might be forced to “explain away” outlier lesions that do not actually harbour the driver mutation. The benefit of biopsy anchoring is conceptually akin to multiple instance learning in other domains, where only a subset of instances in a bag may truly reflect the label [42]. Rather than requiring the model to infer which lesions are representative (a task for which it was not explicitly trained), we perform an upfront curation to supply only representative lesions. This likely helped the model focus on genuine imaging hallmarks associated with biology (for example, texture patterns of EGFR-driven tumours) instead of spurious features needed to fit discordant lesions. In practical terms, our results suggest that when lesion-level labels are available, using them during model training may yield more biologically faithful and robust radiogenomic algorithms.

To our knowledge, this is the first radio(geno)mic study to explicitly compare lesion-level training strategies within the same cohort. The assumption of intra-patient uniformity has generally gone untested in radiogenomics. Most previous studies have either used a single index lesion per patient (e.g., training on primary tumours alone) or pooled all lesions without differentiation. There is growing recognition in medical imaging AI that label noise and annotation granularity can limit model performance [43, 44]. Our work extends that discussion to the area of radiogenomics, highlighting lesion selection as an under-appreciated study design factor.

We also examined the impact of lesion-label fidelity in the context of the frequently cited issue of overfitting in radiomics [45, 46] by performing external validation. Many radiomics models that show high internal accuracy fail to generalise when tested on independent data. In our external cohort (of publicly available data) scanned at different institutions, the biopsy-anchored model proved generalisable, while the more data-rich models could not maintain their advantage outside the training domain. This contrasts with the common assumption that increasing training data improves model performance. While larger datasets can often compensate for imperfect labels, biological heterogeneity can introduce systematic label uncertainty. In such settings, simply increasing the dataset size may amplify noise rather than signal. In our study, adding more lesions introduced instability that became apparent during external validation, suggesting that increasing dataset size without reliable lesion-level labels may not improve radiogenomic model performance. Importantly, this does not imply that smaller curated datasets are universally preferable; rather, dataset scale and label fidelity represent complementary considerations, and their optimal balance may depend on tumour biology, the clinical task, and expert judgement in study design. This study highlights that in radiogenomics, adding more lesions with uncertain labels may not improve model performance when additional cases introduce noise or bias.

In this context, our findings advocate for a shift in how radiogenomic imaging datasets are curated and used. Rather than maximising the number of lesions or voxels regardless of ground-truth confidence, it may be preferable to prioritise lesions with molecular confirmation. This is especially relevant for public repositories and multicentre studies. Including explicit annotation of which lesion was biopsied (as done in our dataset) allows researchers to either train on those lesions or, at the very least, evaluate models with and without them. We recommend that future radiogenomic studies explicitly report their lesion selection strategy and discuss the potential impact of lesion heterogeneity on their models. From a clinical and study design perspective, these findings also have implications for biopsy strategy and dataset curation. Linking imaging features to the specific lesion used for molecular testing may improve the biological fidelity of radiogenomic analyses. When multiple lesions are present, documenting the exact biopsied lesion and preserving this information in imaging datasets could enhance the interpretability and generalisability of downstream AI models. More broadly, our results suggest that future imaging-genomics studies may benefit from incorporating lesion-level ground truth rather than assuming molecular uniformity across all tumour lesions. In cases where multiple lesions are available per patient and biopsy information is lacking, investigators may consider techniques from weakly supervised learning (e.g., multiple instance learning) to allow the model to handle uncertain labels. Alternatively, collecting lesion-specific outcomes through radiogenomic correlative studies or repeat biopsies could greatly enrich modelling (albeit at increased effort). Overall, our results support a more cautious approach to “bulk” labelling of imaging data. Just as genomic studies carefully distinguish between clonal and subclonal mutations, imaging studies should be careful when assuming that all lesions share a single label. By designing datasets and models that account for intra-patient heterogeneity, we can improve reliability and accelerate the translation of radiogenomics into clinical practice.

Non-invasive prediction of tumour mutations has attractive (future) clinical applications. In NSCLC, an imaging-based EGFR assay could be used to triage patients for molecular testing or to flag those who might benefit from a repeat biopsy if initial genotyping is negative despite strong imaging suspicion. It could also guide therapy in scenarios where tissue is hard to sample (such as in unresectable or multifocal disease) or where different lesions in the same patient might carry distinct mutations. An imaging model that identifies divergent radiomic/morphological phenotypes could hypothetically alert clinicians to the presence of multiple primary tumours or mixed responsive/resistant clones. More broadly, radiogenomic models could complement liquid biopsies and other emerging diagnostics by providing a whole-body mutational imaging phenotype. In our study, the features that drive the biopsy-anchored model’s predictions (e.g., texture metrics capturing lesion substructure) are accessible to human interpretation and hypothesis generation. Such insights create opportunities for radiologists to work in tandem with AI, using model outputs and explanations to refine differential diagnoses and select optimal biopsy targets (e.g., choosing a CT target lesion that the model predicts as mutant to increase diagnostic yield).

While the results of this work are encouraging, we acknowledge a few limitations. Given the modest discrimination achieved, the present model should be regarded as exploratory rather than clinically deployable. To minimise overinterpretation, we emphasised generalisability by using patient-aware cross-validation, an internal hold-out set, and independent external validation, and reported uncertainty via bootstrapped confidence intervals. Our primary training cohort was retrospective and drawn from a single institution, which may limit the diversity of imaging characteristics (despite the external validation). Ideally, multicentre trials or prospective studies should confirm that biopsy-anchored radiogenomic modelling retains its advantages across varying scanner types and patient populations. Moreover, we focused on one mutation (EGFR). It remains to be seen whether the observed benefits extend to other genomic alterations or to different types of cancer. Although we carefully segmented the lesions, the biopsy site identification relied on radiology-pathology correspondence. Misclassification of a biopsy site’s lesion could conceivably blunt the benefit of anchoring. We minimised this risk by cross-checking reports and prior scans, but a prospective design with marked biopsy clips could fully mitigate this risk. Additionally, inter-observer variability in lesion segmentation might affect radiomic features. We addressed this by involving an experienced team of radiologists with periodic consensus meetings and by performing quality control on every segmentation. However, even with such safeguards, some degree of variability is inevitable. Prior analyses have shown that many radiomic features can vary with segmentation, especially in heterogeneous tumours [47]. This could have added noise to the modelling, though it impacted all training strategies equally.

Regardless of these limitations, we demonstrate that radiogenomic models trained on biopsy-confirmed lesions can achieve performance comparable to models using all lesions, while offering superior robustness on independent data. In radiogenomics, careful curation of training labels may be more important than increasing dataset size. Future imaging-genomics studies should leverage lesion-level ground truth when available and remain mindful of intrapatient heterogeneity, as embracing these nuances can sharpen the translation of radiomics from petabytes of images to clinically actionable insights.

## Supplementary information

ELECTRONIC SUPPLEMENTARY MATERIAL

## Abbreviations

CE-CT: Contrast-enhanced CT
GLCM: Grey level co-occurrence matrix
GLDM: Grey level dependence matrix
GLRLM: Grey level run length matrix
GLSZM: Grey level size zone matrix
NGS: Next-generation sequencing
NKI: Netherlands Cancer Institute
PACS: Picture Archiving and Communication System
SHAP: SHapley Additive exPlanations
