# Supplementary material for: Quality over quantity: biopsy-anchored CT radiogenomics models outperform all-lesion training in a multi-tumour cohort despite a smaller sample size
Source: Eur Radiol. 2026 May 16;36(10):7473–89. doi: 10.1007/s00330-026-12601-9 (PMC13574897; doi:10.1007/s00330-026-12601-9)

Quality over Quantity: Biopsy-anchored CT Radiogenomics Models Outperform All-lesion Training in a Multi-tumour Cohort Despite Smaller Sample Size

ELECTRONIC SUPPLEMENTARY MATERIAL

Supplementary Material

Supplementary Methods 1. Radiomic feature extraction settings. Full pyradiomics configuration used for feature generation


```
imageType:
  Original:
    binWidth: 5.0
  Square:
    binWidth: 0.9
  SquareRoot:
    binWidth: 13.0
  Logarithm:
    binWidth: 25.0
  Exponential:
    binWidth: 0.1
  Gradient:
    binWidth: 3.0
  LoG:
    binWidth: 3.5
    sigma: [1.0, 2.0, 3.0, 4.0, 5.0]
  Wavelet:
    binWidth: 5.0
  LBP3D:
    binWidth: 1.0

featureClass:
  shape:
  firstorder:
  glcm:
    - 'Autocorrelation'
    - 'JointAverage'
    - 'ClusterProminence'
    - 'ClusterShade'
    - 'ClusterTendency'
    - 'Contrast'
    - 'Correlation'
    - 'DifferenceAverage'
    - 'DifferenceEntropy'
    - 'DifferenceVariance'
    - 'JointEnergy'
    - 'JointEntropy'
    - 'Imc1'
    - 'Imc2'
    - 'Idm'
    - 'Idmn'
```

```
    - 'Id'
    - 'Idn'
    - 'InverseVariance'
    - 'MaximumProbability'
    - 'SumEntropy'
    - 'SumSquares'
  glrlm:
  glszm:
  gldm:
  ngtdm:

setting:
  interpolator: 'sitkBSpline'
  resampledPixelSpacing: [1, 1, 1]
  padDistance: 10
  minimumROIDimensions: 2
  minimumROISize: 1
  correctMask: true

  voxelArrayShift: 1000
  label: 1
```

Supplementary Table 1. Image acquisition parameters. Summary of key CT acquisition characteristics for the NKI and NSCLC-Radiogenomics cohorts. Percentages may not sum to 100% due to rounding.

	**NKI dataset**		**NSCLC-Radiogenomics**	
**Slice Thickness [mm]**	1.0 (1.0–1.5)		1.2 (1.0–1.2)	
**KVP [kV]**	120.0 (120.0–120.0)		120.0 (120.0–120.0)	
**Tube Current [mAs]**	182.0 (139.0–294.2)		354.5 (244.5–519.0)	
**Exposure [mAs]**	96.0 (73.0–170.0)		6.5 (3.0–208.8)	
**Pixel Spacing [mm]**	0.782 (0.726–0.839)		0.777 (0.703–0.820)	
**Scanner model**				
	Canon Aquilion	919 (55.8%)	GE Discovery CT750 HD	64 (40.5%)
	Siemens SOMATOM Sensation Open	404 (24.5%)	GE LightSpeed VCT	7 (4.4%)
	Siemens SOMATOM Confidence	149 (9.1%)	Others	3 (1.9%)
	Philips GEMINI TF TOF 16	66 (4.0%)	Missing	84 (53.2%)
	Philips Vereos PET/CT	24 (1.5%)		
	Others	84 (5.1%)		

Supplementary Table 2. Training cohort-specific characteristics. Lesion-level descriptors across the all-lesion, non-biopsied, and biopsied lesion training cohorts.

	All lesions	Non-biopsied lesions	Biopsied lesions
**Tumour volume [mm3] (median, IQR)**	1412.9 [1341.5–1479.2]	1197.7 [1137.3–1257.8]	15783.3 [12943.9–19189.5]
**Tumour types (n, %)**			
Lung cancer	3676 (39.9%)	3360 (39.7%)	316 (41.7%)
Colorectal cancer	2258 (24.5%)	2117 (25.0%)	141 (18.6%)
Others	3288 (35.7%)	2988 (35.3%)	300 (39.6%)
**Lesion location (n, %)**			
Lung	2280 (24.7%)	2079 (24.6%)	201 (26.6%)
Liver	1966 (21.3%)	1860 (22.0%)	106 (14.0%)
Lymph node	2241 (24.3%)	2153 (25.4%)	88 (11.6%)
Bone	884 (9.6%)	847 (10.0%)	37 (4.9%)
Other	1851 (20.1%)	1526 (18.0%)	325 (42.9%)

Supplementary Table 3. Selected features per training cohort. Radiomic features chosen by the ensemble selector for each training cohort.

All lesions	Non-biopsied lesions	Biopsied lesions
Square GLCM JointAverage	Square GLCM JointAverage	Wavelet-LLH GLSZM SZNUN
Square FirstOrder Median	Square FirstOrder Median	LoG_σ_1 GLRLM RunEntropy
Original GLDM DNUN	Wavelet-LLH GLSZM ZonePercentage	LoG_σ_1 GLDM DependenceEntropy
Wavelet-LLH GLSZM ZonePercentage	Wavelet-HLH GLCM ClusterShade	
Wavelet-LLH FirstOrder 90Percentile	Square GLSZM SZNUN	
Wavelet-HLL FirstOrder Skewness	Square GLCM LMC1	
LoG_σ_5 FirstOrder Skewness	Original GLDM DNUN	
LoG_σ_5 FirstOrder Kurtosis	Log GLSZM SAE	
Exp GLDM DNUN	LoG_σ_1 GLSZM ZonePercentage	
	LBP GLCM ClusterShade	
	LBP GLSZM LGLZE	
	Exp GLDM DNUN	

Supplementary Table 4. Shapiro-Wilk test for normality. Normality testing of selected radiomic features in the training set, confirming non-Gaussian distributions and supporting the use of non-parametric statistics in univariate analyses.

Feature name	Training cohort	Shapiro statistic	p	Adjusted p (q)
Exp GLDM DNUN	All lesion	0.75	<0.001	**<0.001**
LoG_σ_5 FirstOrder Kurtosis	All lesion	0.33	<0.001	**<0.001**
LoG_σ_5 FirstOrder Skewness	All lesion	0.88	<0.001	**<0.001**
Original GLDM DNUN	All lesion	0.78	<0.001	**<0.001**
Square FirstOrder Median	All lesion	0.31	<0.001	**<0.001**
Square GLCM JointAverage	All lesion	0.34	<0.001	**<0.001**
Wavelet-HLL FirstOrder Skewness	All lesion	0.73	<0.001	**<0.001**
Wavelet-LLH FirstOrder 90Percentile	All lesion	0.63	<0.001	**<0.001**
Wavelet-LLH GLSZM ZonePercentage	All lesion	0.95	<0.001	**<0.001**
LoG_σ_1 GLDM DependenceEntropy	Biopsied lesions	0.96	<0.001	**<0.001**
LoG_σ_1 GLRLM RunEntropy	Biopsied lesions	0.89	<0.001	**<0.001**
Wavelet-LLH GLSZM SZNUN	Biopsied lesions	0.9	<0.001	**<0.001**
Exp GLDM DNUN	Non-biopsied lesions	0.76	<0.001	**<0.001**
LBP GLSZM LGLZE	Non-biopsied lesions	0.99	<0.001	**<0.001**
LBP GLCM ClusterShade	Non-biopsied lesions	0.97	<0.001	**<0.001**
LoG_σ_1 GLSZM ZonePercentage	Non-biopsied lesions	0.9	<0.001	**<0.001**
Log GLSZM SAE	Non-biopsied lesions	0.87	<0.001	**<0.001**
Original GLDM DNUN	Non-biopsied lesions	0.79	<0.001	**<0.001**
Square FirstOrder Median	Non-biopsied lesions	0.33	<0.001	**<0.001**
Square GLCM LMC1	Non-biopsied lesions	0.78	<0.001	**<0.001**
Square GLCM JointAverage	Non-biopsied lesions	0.35	<0.001	**<0.001**
Square GLSZM SZNUN	Non-biopsied lesions	0.92	<0.001	**<0.001**
Wavelet-HLH GLCM ClusterShade	Non-biopsied lesions	0.25	<0.001	**<0.001**
Wavelet-LLH GLSZM ZonePercentage	Non-biopsied lesions	0.95	<0.001	**<0.001**

Supplementary Table 5. Classifiers and hyperparameters using all available lesions. Final TPOT-selected pipelines and settings for the three full-cohort models, listing the chosen classifier and the fitted hyperparameters for each training strategy.

Training cohort	EGFR Classifier and hyperparameters
All lesions	XGBClassifier {'objective': 'binary:logistic', 'base_score': None, 'booster': None, 'callbacks': None, 'colsample_bylevel': None, 'colsample_bynode': None, 'colsample_bytree': 0.8, 'device': None, 'early_stopping_rounds': None, 'enable_categorical': False, 'eval_metric': 'logloss', 'feature_types': None, 'feature_weights': None, 'gamma': None, 'grow_policy': None, 'importance_type': None, 'interaction_constraints': None, 'learning_rate': 0.01, 'max_bin': None, 'max_cat_threshold': None, 'max_cat_to_onehot': None, 'max_delta_step': None, 'max_depth': 5, 'max_leaves': None, 'min_child_weight': 5, 'missing': nan, 'monotone_constraints': None, 'multi_strategy': None, 'n_estimators': 100, 'n_jobs': None, 'num_parallel_tree': None, 'random_state': 42, 'reg_alpha': None, 'reg_lambda': None, 'sampling_method': None, 'scale_pos_weight': None, 'subsample': 0.8, 'tree_method': None, 'validate_parameters': None, 'verbosity': 0, 'use_label_encoder': False}
Non-biopsied lesions	XGBClassifier {'objective': 'binary:logistic', 'base_score': None, 'booster': None, 'callbacks': None, 'colsample_bylevel': None, 'colsample_bynode': None, 'colsample_bytree': 0.8, 'device': None, 'early_stopping_rounds': None, 'enable_categorical': False, 'eval_metric': 'logloss', 'feature_types': None, 'feature_weights': None, 'gamma': None, 'grow_policy': None, 'importance_type': None, 'interaction_constraints': None, 'learning_rate': 0.01, 'max_bin': None, 'max_cat_threshold': None, 'max_cat_to_onehot': None, 'max_delta_step': None, 'max_depth': 5, 'max_leaves': None, 'min_child_weight': 5, 'missing': nan, 'monotone_constraints': None, 'multi_strategy': None, 'n_estimators': 100, 'n_jobs': None, 'num_parallel_tree': None, 'random_state': 42, 'reg_alpha': None, 'reg_lambda': None, 'sampling_method': None, 'scale_pos_weight': None, 'subsample': 0.8, 'tree_method': None, 'validate_parameters': None, 'verbosity': 0, 'use_label_encoder': False}
Biopsied lesions	XGBClassifier {'objective': 'binary:logistic', 'base_score': None, 'booster': None, 'callbacks': None, 'colsample_bylevel': None, 'colsample_bynode': None, 'colsample_bytree': 0.8, 'device': None, 'early_stopping_rounds': None, 'enable_categorical': False, 'eval_metric': 'logloss', 'feature_types': None, 'feature_weights': None, 'gamma': None, 'grow_policy': None, 'importance_type': None, 'interaction_constraints': None, 'learning_rate': 0.01, 'max_bin': None, 'max_cat_threshold': None, 'max_cat_to_onehot': None, 'max_delta_step': None, 'max_depth': 3, 'max_leaves': None, 'min_child_weight': 5, 'missing': nan, 'monotone_constraints': None, 'multi_strategy': None, 'n_estimators': 100, 'n_jobs': None, 'num_parallel_tree': None, 'random_state': 42, 'reg_alpha': None, 'reg_lambda': None, 'sampling_method': None, 'scale_pos_weight': None, 'subsample': 0.8, 'tree_method': None, 'validate_parameters': None, 'verbosity': 0, 'use_label_encoder': False}

Supplementary Table 6. Multivariate analysis of model predictions. Logistic regression assessing associations between predicted EGFR probability and mutational status while adjusting for lesion volume and tumour type, reported separately for all-lesion, non-biopsied, and biopsied models.

	**All lesions**				**Non-biopsied lesions**				**Biopsied lesions**			
	**Coefficient (95% CI)**	**Std. error**	**z**	**p**	**Coefficient (95% CI)**	**Std. error**	**z**	**p**	**Coefficient (95% CI)**	**Std. error**	**z**	**p**
**Intercept**	-9.46 (-13.09--5.84)	1.85	-5.11	**<0.001**	-9.40 (-13.02--5.77)	1.85	-5.09	**<0.001**	-9.00 (-12.65--5.35)	1.86	-4.83	**<0.001**
**AI scores**	4.59 (3.30-5.87)	0.66	6.99	**<0.001**	4.46 (3.25-5.68)	0.62	7.20	**<0.001**	3.16 (1.74-4.58)	0.72	4.37	**<0.001**
**Volume**	-0.04 (-0.35-0.27)	0.16	-0.26	0.796	-0.03 (-0.34-0.28)	0.16	-0.21	0.836	-0.09 (-0.44-0.26)	0.18	-0.50	0.617
**Tumour type: lung**	6.13 (2.55-9.72)	1.83	3.36	**0.001**	6.17 (2.59-9.75)	1.83	3.38	**0.001**	6.28 (2.70-9.86)	1.83	3.44	**0.001**
**Tumour type: Other**	3.34 (-0.27-6.96)	1.85	1.81	0.070	3.37 (-0.25-6.98)	1.85	1.82	0.068	3.43 (-0.18-7.05)	1.85	1.86	0.063

Supplementary Table 7. Classifiers and hyperparameters for size-matched all-lesion sub-samples. TPOT-selected classifiers and hyperparameters for the ten sub-samples of the all-lesion cohort.

Training cohort	Sample	Classifier and hyperparameters
All lesions	**Sub-sample 1**	LinearSVC(C=0.005994842503189409, class_weight='balanced',dual=False, random_state=42)
	**Sub-sample 2**	LogisticRegression(C=0.005994842503189409,class_weight='balanced', random_state=42,solver='liblinear')
	**Sub-sample 3**	XGBClassifier(base_score=None, booster=None, callbacks=None,colsample_bylevel=None, colsample_bynode=None,colsample_bytree=0.8, device=None,early_stopping_rounds=None,enable_categorical=False, eval_metric='logloss',feature_types=None, feature_weights=None,gamma=None, grow_policy=None,importance_type=None,interaction_constraints=None, learning_rate=0.01,max_bin=None, max_cat_threshold=None,max_cat_to_onehot=None, max_delta_step=None,max_depth=3, max_leaves=None, min_child_weight=5,missing=nan, monotone_constraints=None,multi_strategy=None, n_estimators=100,n_jobs=None, num_parallel_tree=None, ...)
	**Sub-sample 4**	LogisticRegression(C=0.0001, class_weight='balanced',random_state=42, solver='liblinear')
	**Sub-sample 5**	LogisticRegression(C=0.0001, class_weight='balanced',random_state=42, solver='liblinear')
	**Sub-sample 6**	LogisticRegression(C=10000.0, class_weight='balanced',random_state=42, solver='liblinear')
	**Sub-sample 7**	LGBMClassifier(colsample_bytree=0.8, learning_rate=0.01,max_depth=10, random_state=42,subsample=0.8)
	**Sub-sample 8**	LogisticRegression(C=0.005994842503189409, random_state=42,solver='liblinear'
	**Sub-sample 9**	LogisticRegression(C=0.000774263682681127,class_weight='balanced', random_state=42,solver='liblinear')

	**Sub-sample 10**	LinearSVC(C=166.81005372000558, dual=False, random_state=42)

Supplementary Table 8. Classifier and hyperparameters for the sized-matched groups of the non-biopsied lesion training cohort. TPOT-selected pipelines for the ten sub-samples of the non-biopsied cohort, listing the chosen classifier and corresponding hyperparameters for each sub-sample.

**Non-biopsied lesions**	**Sub-sample 1**	GradientBoostingClassifier(learning_rate=0.01,random_state=42)
	**Sub-sample 2**	LogisticRegression(C=0.046415888336127774,class_weight='balanced', random_state=42,solver='liblinear')
	**Sub-sample 3**	LinearSVC(C=0.005994842503189409, class_weight='balanced',dual=False, random_state=42)
	**Sub-sample 4**	LogisticRegression(C=0.046415888336127774, random_state=42,solver='liblinear')
	**Sub-sample 5**	XGBClassifier(base_score=None, booster=None, callbacks=None,colsample_bylevel=None, colsample_bynode=None,colsample_bytree=0.8, device=None,early_stopping_rounds=None,enable_categorical=False, eval_metric='logloss',feature_types=None, feature_weights=None,gamma=None, grow_policy=None,importance_type=None,interaction_constraints=None, learning_rate=0.01,max_bin=None, max_cat_threshold=None,max_cat_to_onehot=None, max_delta_step=None,max_depth=3, max_leaves=None, min_child_weight=5,missing=nan, monotone_constraints=None,multi_strategy=None, n_estimators=100,n_jobs=None, num_parallel_tree=None, ...)
	**Sub-sample 6**	LinearSVC(C=0.046415888336127774, dual=False,random_state=42)
	**Sub-sample 7**	LogisticRegression(C=0.0001, random_state=42,solver='liblinear')
	**Sub-sample 8**	LinearSVC(C=0.046415888336127774, class_weight='balanced',dual=False, random_state=42)
	**Sub-sample 9**	LinearSVC(C=0.000774263682681127, dual=False,random_state=42)

	**Sub-sample 10**	LogisticRegression(C=1291.5496650148827,class_weight='balanced', random_state=42,solver='liblinear')

Supplementary Table 9. Clinical characteristics of size-matched training sub-samples. Patient and lesion counts and EGFR mutation rates for each of the ten sub-samples in the all-lesion and non-biopsied size-matched analyses, with the all-lesion sub-samples additionally reporting the number of biopsied lesions included.

Training cohort	Sample	Patients (n)	Lesions (n)	Biopsied lesions (n)	EGFR mutation rate (%)
All lesions	**Sub-sample 1**	757	757	190	9.91%
	**Sub-sample 2**	757	757	73	8.85%
	**Sub-sample 3**	757	757	167	10.04%
	**Sub-sample 4**	757	757	184	9.64%
	**Sub-sample 5**	757	757	181	8.72%
	**Sub-sample 6**	757	757	180	10.17%
	**Sub-sample 7**	757	757	168	8.59%
	**Sub-sample 8**	757	757	182	9.38%
	**Sub-sample 9**	757	757	190	10.57%
	**Sub-sample 10**	757	757	178	9.91%
Non-biopsied lesions	**Sub-sample 1**	757	757	-	10.04%
	**Sub-sample 2**	757	757	-	10.57%
	**Sub-sample 3**	757	757	-	10.44%
	**Sub-sample 4**	757	757	-	10.57%
	**Sub-sample 5**	757	757	-	11.49%
	**Sub-sample 6**	757	757	-	10.96%
	**Sub-sample 7**	757	757	-	11.10%
	**Sub-sample 8**	757	757	-	10.44%
	**Sub-sample 9**	757	757	-	10.70%
	**Sub-sample 10**	757	757	-	10.83%

Supplementary Table 10. Performance internal test consisting of only biopsied lesions. Evaluation of the three full-cohort models on the internal validation set restricted to only biopsied lesions (n=181; 18 EGFR-positive).

Training cohort	Validation	ROC-AUC	F1-score	Accuracy	PPV	NPV	p
All lesions	Internal (only biopsied)	0.53 [0.38-0.67]	0.06 [0.00-0.21]	0.85 [0.80-0.90]	0.08 [0.00-0.29]	0.90 [0.86-0.94]	0.68
Biopsied	Internal (only biopsied)	0.67 [0.55-0.79]	0.26 [0.13-0.38]	0.69 [0.62-0.76]	0.17 [0.08-0.27]	0.94 [0.89-0.97]	**0.02**
Non-biopsied	Internal (only biopsied)	0.56 [0.40-0.71]	0.08 [0.00-0.25]	0.87 [0.83-0.92]	0.12 [0.00-0.50]	0.90 [0.86-0.94]	0.39

Supplementary Figure 1. Feature correlation in the all-lesion signature. Heatmap of (absolute) pairwise correlations among the features selected for the all-lesion model.

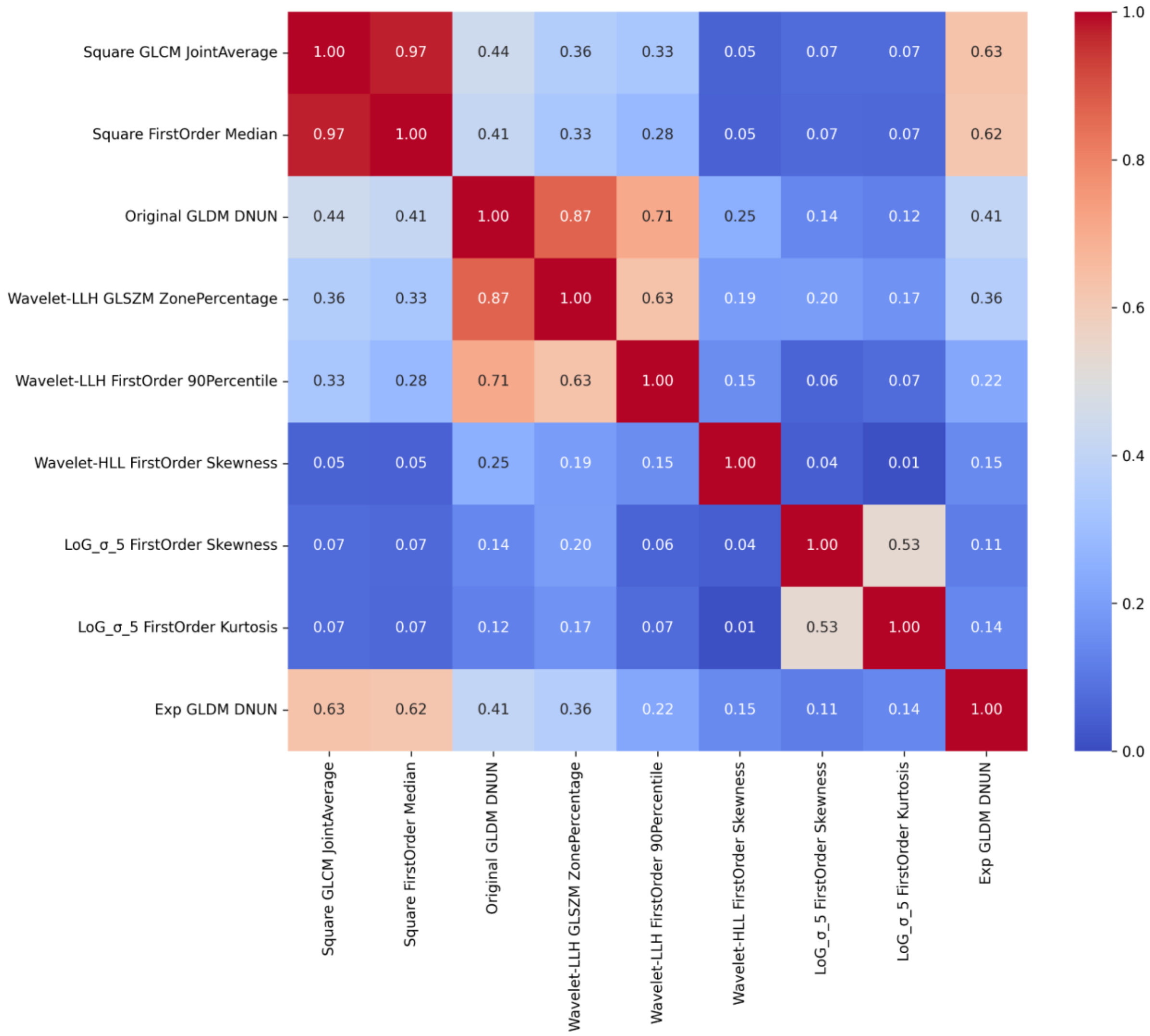

Supplementary Figure 2. Feature correlation in the non-biopsied signature. Heatmap of pairwise Pearson correlations among the features selected for the non-biopsied lesion model.

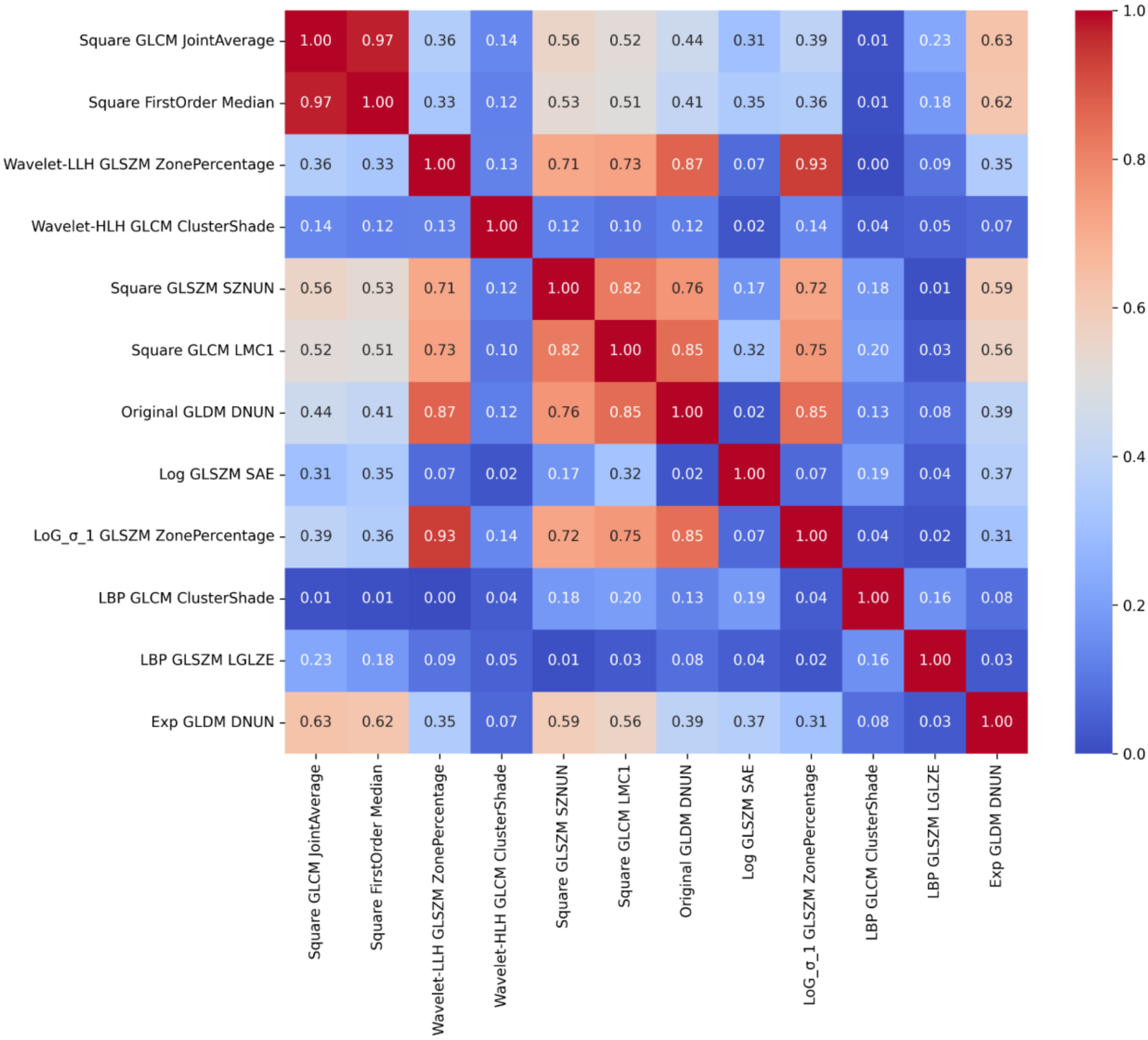

Supplementary Figure 3. Feature correlation in the biopsied-lesion signature. Heatmap of pairwise Pearson correlations among the features selected for the biopsied-lesion model.

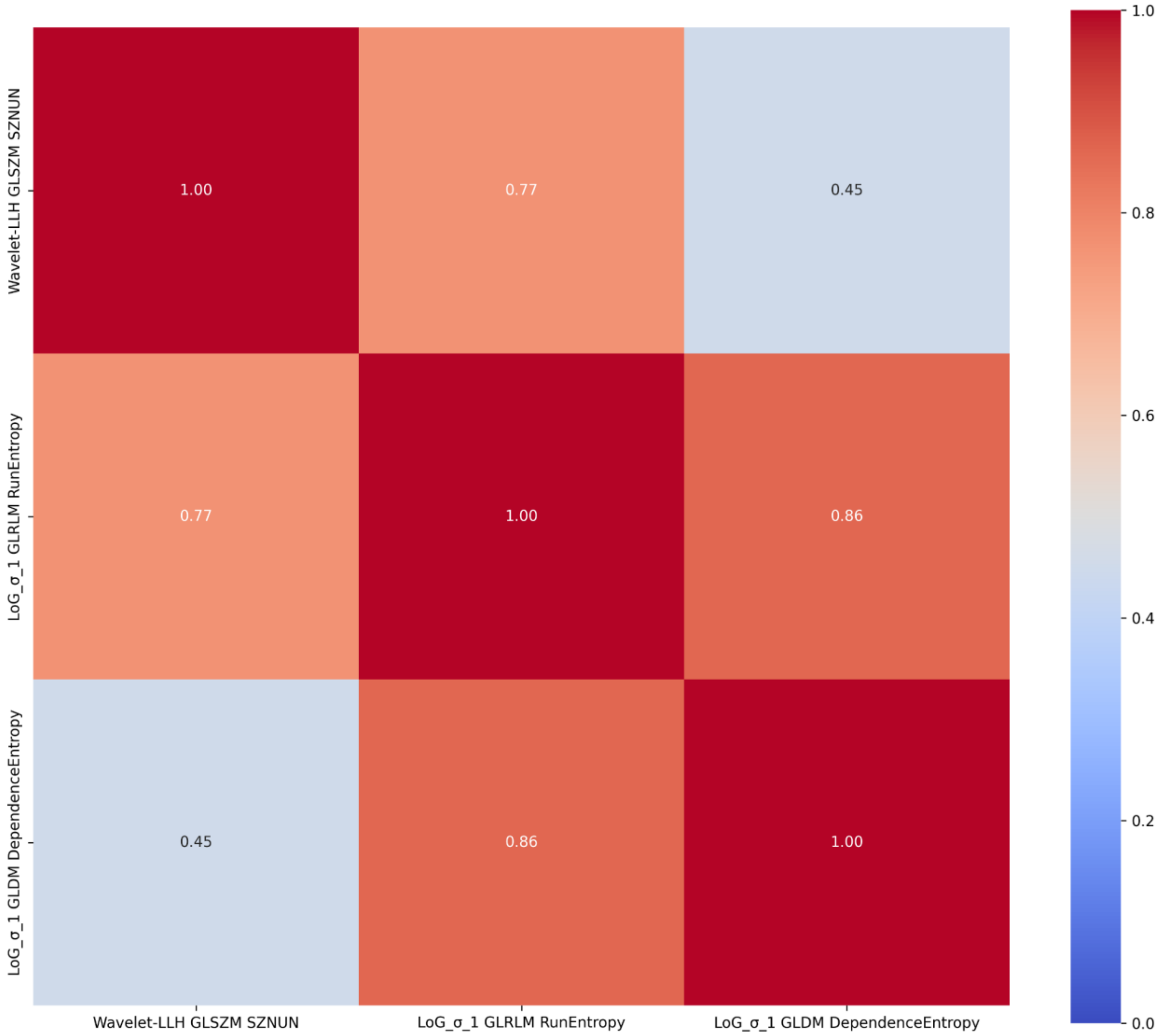

Supplement: Supplementary file 1 [file 330_2026_12601_MOESM1_ESM.pdf]
